# Bibliometric analysis of scientific papers on extracellular vesicles in kidney disease published between 1999 and 2022

**DOI:** 10.3389/fcell.2022.1070516

**Published:** 2023-01-05

**Authors:** Marady Hun, Huai Wen, Phanna Han, Tharith Vun, Mingyi Zhao, Qingnan He

**Affiliations:** ^1^ Department of Pediatrics, The Third Xiangya Hospital, Central South University, Changsha, China; ^2^ Department of Ophthalmology, The Second Xiangya Hospital, Central South University, Changsha, China; ^3^ Department of General Surgery, Xiangya Hospital, Central South University, Changsha, China

**Keywords:** kidney disease, EVs, exosomes, acute kidney disease, chronic kidney disease, bibliometric analysis

## Abstract

**Background:** In recent years, there has been an increasing interest in using extracellular vesicles (EVs) as potential therapeutic agents or natural drug delivery systems in kidney-related diseases. However, a detailed and targeted report on the current condition of extracellular vesicle research in kidney-related diseases is lacking. Therefore, this prospective study was designed to investigate the use of bibliometric analysis to comprehensively overview the current state of research and frontier trends on extracellular vesicle research in kidney-related diseases using visualization tools.

**Methods:** The Web of Science Core Collection (WoSCC) database was searched to identify publications related to extracellular vesicle research in kidney-related diseases since 1999. Citespace, Microsoft Excel 2019, VOSviewer software, the R Bibliometrix Package, and an online platform were used to analyze related research trends to stratify the publication data and collaborations.

**Results:** From 1 January 1999 to 26 June 2022, a total of 1,122 EV-related articles and reviews were published, and 6,486 authors from 1,432 institutions in 63 countries or regions investigated the role of extracellular vesicles in kidney-related diseases. We found that the number of articles on extracellular vesicles in kidney-related diseases increased every year. Dozens of publications were from China and the United States. China had the most number of related publications, in which the Southeast University (China) was the most active institution in all EV-related fields. Liu Bi-cheng published the most papers on extracellular vesicles, while Clotilde Théry had the most number of co-citations. Most papers were published by The International Journal of Molecular Sciences, while Kidney International was the most co-cited journal for extracellular vesicles. We found that exosome-related keywords included exosome, exosm, expression, extracellular vesicle, microRNA, microvesicle, and liquid biopsy, while disease- and pathological-related keywords included biomarker, microRNA, apoptosis, mechanism, systemic lupus erythematosus, EGFR, acute kidney injury, and chronic kidney disease. Acute kidney disease (AKI), CKD, SLE, exosome, liquid biopsy, and extracellular vesicle were the hotspot in extracellular vesicle and kidney-related diseases research.

**Conclusion:** The field of extracellular vesicles in kidney-related disease research is rapidly growing, and its domain is likely to expand in the next decade. The findings from this comprehensive analysis of extracellular vesicles in kidney-related disease research could help investigators to set new diagnostic, therapeutic, and prognostic ideas or methods in kidney-related diseases.

## Introduction

Kidney-related diseases, i.e., acute or chronic illnesses, have a significant influence on global health as a direct cause of morbidity and mortality (GBD Chronic Kidney Disease Collaboration, 2020; Kellum et al., 2021). To our knowledge, acute kidney disease (AKI) is generally acknowledged as the main risk factor for the incidence and mortality of chronic kidney disease (CKD). Short- and long-term status significantly affects functional conditions and may exacerbate resource utilization (Bellomo et al., 2012; Levey et al., 2015; Kellum et al., 2021). AKI-related mortality by far surpasses the mortality of other diseases, such as diabetes, breast cancer, and heart failure (Lewington et al., 2013; GBD Chronic Kidney Disease Collaboration, 2020; Kellum et al., 2021; Murray et al., 2021). According to the Global Burden of Diseases (GBD) Chronic Kidney Disease Collaboration statistics, ∼1.2 million people died from CKD in 2017, and the global all-age mortality rate from CKD increased by 41.5% between 1990 and 2017. The report from the Chronic Kidney Disease surveillance system in the United States in 2018 suggested that ∼37 million adult Americans, i.e., 15% of the US population, were affected by CKD, and 1 in every 3 US adults could develop CKD (Murray et al., 2021). Moreover, CKD-related cardiovascular disease was reported in 2.6 million deaths and 35.8 million disability-adjusted life-years (DALYs), with most DALYs attributable to CKD occurring in middle and low-middle socio-demographic index (SDI) quintiles. However, since CKD is preventable and treatable, it is worth paying more attention to its global health policy decision-making, especially in locations with low and middle SDI (GBD Chronic Kidney Disease Collaboration, 2020; Vanholder et al., 2022). According to these reports, we found a lack of improvement in the diagnosis and treatment of kidney-related diseases, leading to unmet clinical or medical needs. Therefore, there is an imperative requirement for new perspectives to treat kidney disease and its related complications or other burdens.

Due to the global burden and significant influence of an ever-increasing risk of kidney diseases (Bellomo et al., 2012; GBD Chronic Kidney Disease Collaboration, 2020; Kellum et al., 2021), there has been an increasing need for improving new therapeutics to control these diseases. In a few previous studies about EVs in kidney-related disease (Pisitkun et al., 2004; Zhou et al., 2006a; Bruno et al., 2009; Gonzales et al., 2009; Miranda et al., 2010; van Balkom et al., 2011; Alvarez et al., 2012; Merino et al., 2014; Fais et al., 2016), it was shown that EVs are endogenous or natural membranous nanoparticles that play important roles in essentially all organisms; were used as drug carriers in nanomedicine; and were found to play important roles in the diagnosis, treatment, and prognosis of kidney-related diseases (Kalluri and LeBleu, 2020; Lu and Huang, 2020; Fan et al., 2022; Tang et al., 2022; Yang et al., 2022). Recently, there have been increasing developments on EVs for the detection and treatment of more different types of kidney diseases, including CKD (Sun et al., 2019; Tang et al., 2020a; Grange et al., 2020; Jin et al., 2021; Grange and Bussolati, 2022; Tang et al., 2022; Wang et al., 2022), AKI (Sun et al., 2019; Tang et al., 2020a; Xie et al., 2020; Jin et al., 2021; Kraińska et al., 2021; Grange and Bussolati, 2022; Tang et al., 2022), lupus nephritis (Thongboonkerd, 2019; Garcia-Vives et al., 2020; Xie et al., 2020; van Zonneveld et al., 2021; Grange and Bussolati, 2022), kidney-related inflammation (Sun et al., 2019; Thongboonkerd, 2019; Tang et al., 2020a; Tang et al., 2020b; van Zonneveld et al., 2021), glomerular-related disorders (Thongboonkerd, 2019; Jin et al., 2021; Grange and Bussolati, 2022), transplantation (Thongboonkerd, 2019; Grange and Bussolati, 2022), diabetes (Thongboonkerd, 2019; Grange and Bussolati, 2022; Tang et al., 2022), cancer (Thongboonkerd, 2019; van Zonneveld et al., 2021), fibrosis (Thongboonkerd, 2019; Jin et al., 2020; Tang et al., 2022; Wang et al., 2022), drug resistance (Li et al., 2021; van Zonneveld et al., 2021), toxicity (Jiang et al., 2020), focal segmental glomerulosclerosis (Grange and Bussolati, 2022; Tang et al., 2022), and IgA nephropathy (Grange and Bussolati, 2022). However, despite the increased comprehension of the detection and treatment of EVs in kidney-related diseases (Lewington et al., 2013; Tang et al., 2020a; Jiang et al., 2020; van Zonneveld et al., 2021; Grange and Bussolati, 2022), with the rapid rise in the number of related publications, it is becoming increasingly difficult for scholars to summarize/catch up with the latest discoveries in this field. Thus, in this study, we conducted bibliometrics research on EVs in kidney-related diseases based on existing literature to provide a comprehensive overview of current hotspots for better decision-making in this domain.

## Materials and methods

### Data sources and search strategies

**FIGURE 1:**
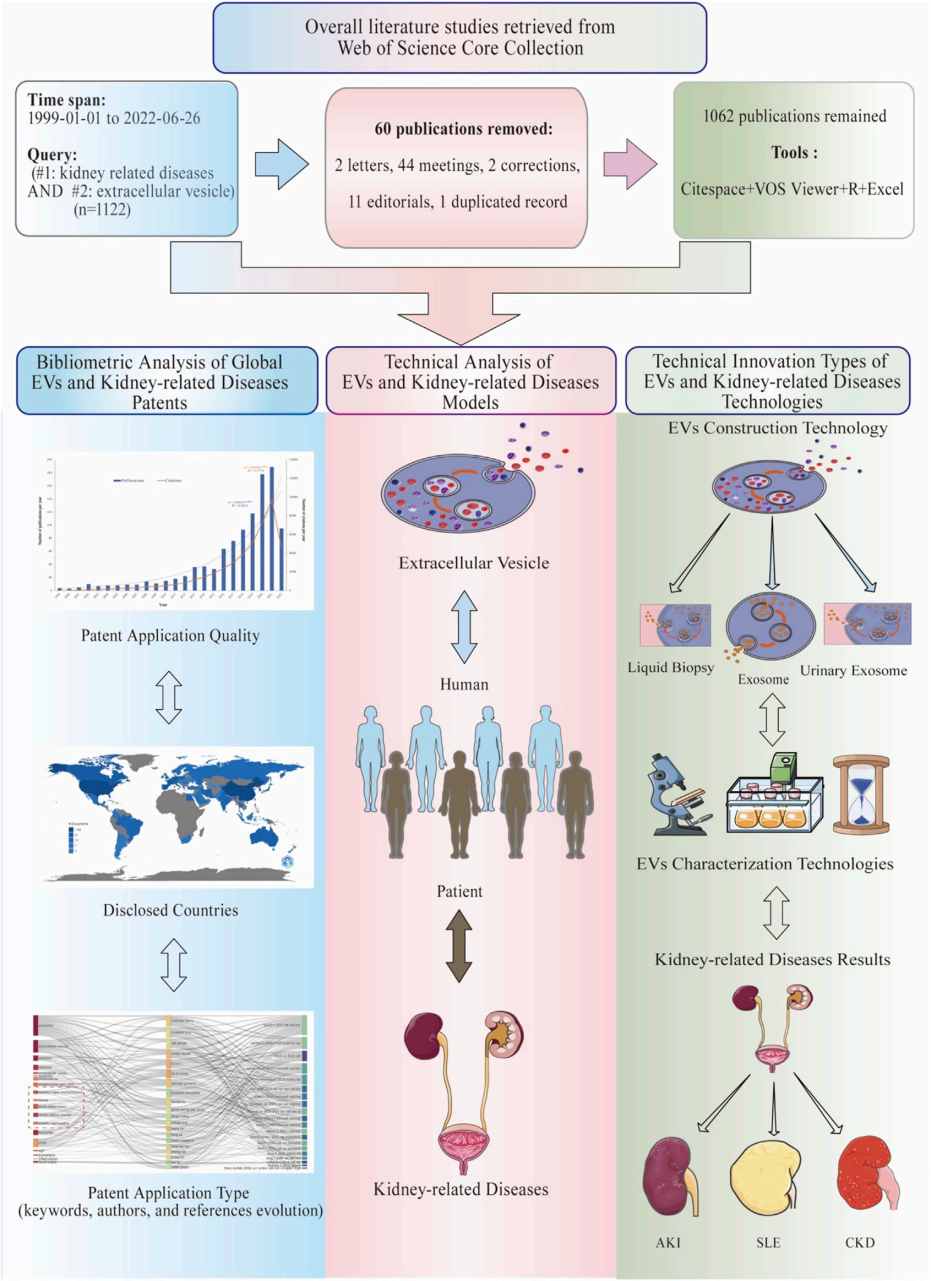
Flow diagram of the included publications, methods, and results of bibliometric analysis.

The Web of Science Core Collection (WoSCC) database was searched to obtain data on EVs in kidney-related diseases over the past 22 years (from 1 January 1999 to 26 June 2022). The search formula was set as [TS = (extracellular vesicle) AND TS = (kidney-related diseases)]. In this context, we selected a dataset derived from WoSCC as our target dataset for analytic purposes. Since the topic search of WoSCC can be interpreted as a model for keyword search based on words in the title, abstract, author keywords, and keyword plus, we chose the search topic to obtain a more precise topic. Moreover, we used MeSH to extract all search samples. A literature review was conducted on the following topics: the whole search formula of [TS = (extracellular vesicle) AND TS = (kidney-related diseases)] was set as [TS = (“extracellular vesicle” OR “vesicle, extracellular” OR “vesicles, extracellular” OR “exovesicles” OR “exovesicle” OR “exosomes” OR “apoptotic bodies” OR “apoptotic body” OR “bodies, apoptotic” OR “body, apoptotic”)] AND [TS = (“chronic kidney failure” OR “chronic renal insufficiency” OR “chronic kidney disease*” OR “renal failure*” OR “kidney failure” OR “renal impairment” OR “kidney impairment” OR “kidney dysfunction” OR “renal dysfunction” OR “reduced renal function” OR “CKD” OR “progressive kidney” OR “glomerular filtration rate” OR “GFR” OR “eGFR” OR proteinuri* OR “albuminuria” OR “microalbuminuria” OR “end-stage renal disease” OR “ESRD” OR “end-stage kidney disease” OR “ESKD” OR “dialysis” OR “renal replacement therapy” OR “kidney transplant” OR “lupus nephritis” OR “acute kidney injuries” OR “acute renal injury” OR “acute renal injuries” OR “acute renal insufficiencies” OR “acute kidney insufficiencies” OR “acute kidney insufficiency” OR “acute renal failure” OR “acute renal failures” OR “AKI” OR “systemic lupus erythematosus” OR “SLE” OR “nephrotic syndrome” OR “NS” OR “glomerulonephritides” OR “iga glomerulonephritis” OR “iga nephropathy” OR “nephritis”)]. The following information was gathered for the inclusion of related studies: the number of publications and citations, titles, publication year, countries, affiliations, authors, journals, keywords, and references of each publication (Figure 1; Supplementary Table S1).

### Statistical analysis

For data analysis and visualization, it is necessary to have the appropriate software and online platform. Full records and cited references of all the documents in txt format were downloaded and assembled from WoSCC and imported to CiteSpace 6.1R2, 64 bits basic (Drexel University, Philadelphia, PA, United States), VOSviewer 1.6.17 (Leiden University, Netherlands), Microsoft Excel 2019, the R Bibliometrix Package, and one online platform (https://bibliometric.com) (Figure 1).

CiteSpace, a freely available Java application by Chen (2004), is widely used to analyze and visualize trends and designs in the scientific literature (Synnestvedt et al., 2005), with WoSCC as its main input data source. For this study, we performed clustering, timeline, and reference burst analysis of EVs in kidney-related diseases using CiteSpace.

VOSviewer software (https://www.vosviewer.com/) is an important and powerful tool for building and visualizing bibliometric networks (van Eck and Waltman, 2010). Co-authorship and citation-based and co-occurrence networks could be established based on data downloaded from the Web of Science. In this study, the co-citation networks examined were analyzed to identify countries, institutions, and authors that collaborated and journals that mutually cited one another. Co-occurrence networks were used to investigate keyword occurrences across publications. The size of the nodes represents publication numbers, and the thickness of the connecting lines provides a measurement of each node’s strength. Co-citation and co-occurrence networks were presented in separate parts for the threshold (the minimum number of documents and the maximum link strength) associated with each of these networks. An item’s circle size was proportional to its number of publications, and its line width was proportional to the magnitude of its links. An item’s total link strength reflects how closely the item was associated with others, while the same color indicated close association.

The R language-based Bibliometrix Package (4.1.0 Package), which can show publications of the 10 most productive authors over the past 22 years and identify research topic evolution, was used to analyze country scientific production, three-field plots (co-cited references, authors, and keywords), author production over time, author impact (H-index), and thematic evolution analysis.

In addition to the aforementioned software, assessments on cooperation relationships among countries were also performed on bibliometric analysis software (https://bibliometric.com/).

## Results

### Analysis of the publication and citation trends

**FIGURE 2:**
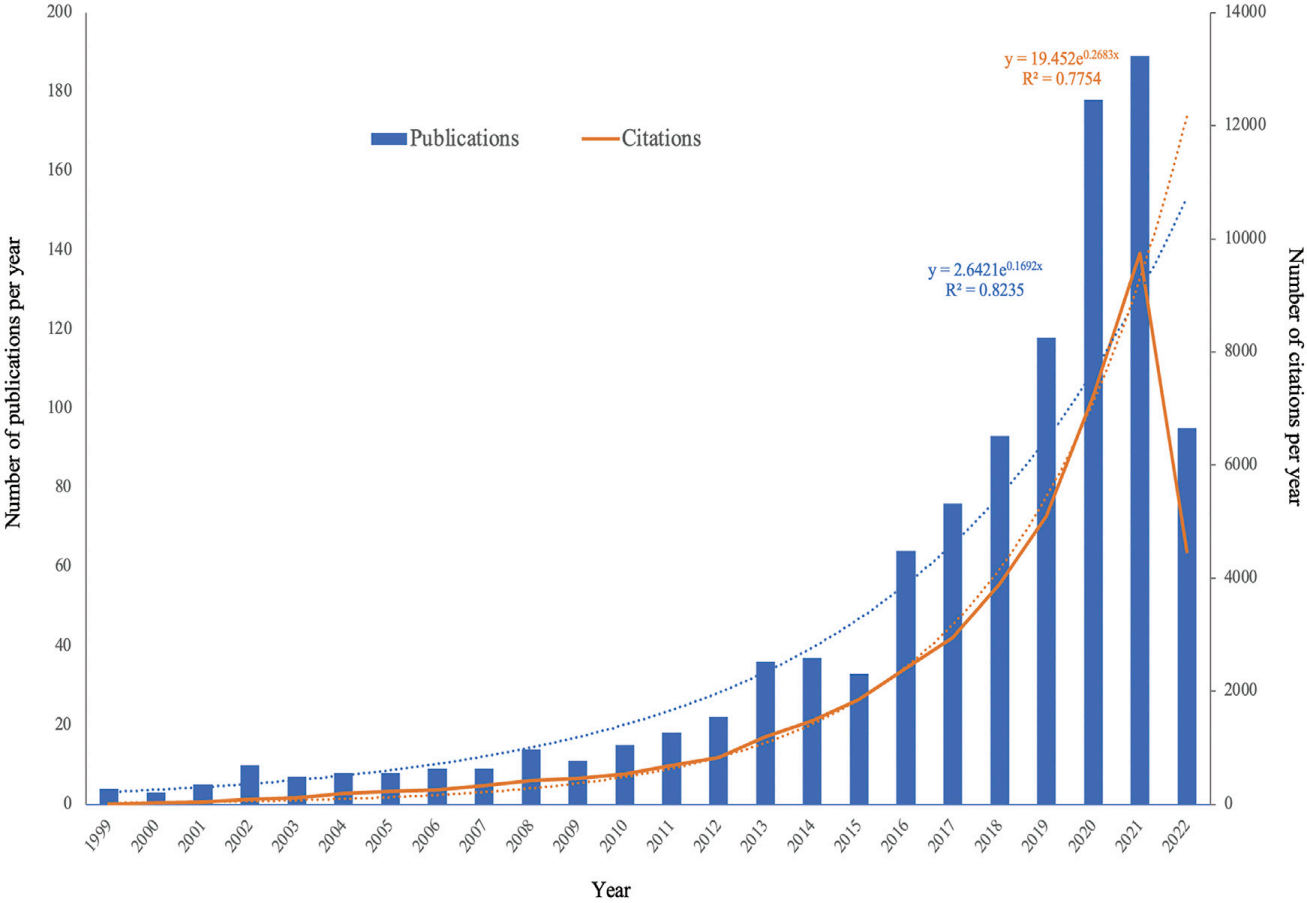
Annual publication and annual citation trends in the past 22 years.

A total of 1,122 studies were obtained based on our search strategy. After excluding 60 articles, comprising meetings (*n* = 44), letters (*n* = 2), editorials (*n* = 11), corrections (*n* = 2), and duplicated records (*n* = 1), 1,062 articles in English were found eligible for this present study. We found a significant increase in global publications in the field, from four publications in 1999 to 189 publications in 2021. The specific search formula and sample are shown in Figure 1 and Supplementary Table S1A. Figure 2 and Supplementary Table S1B show that the number of EV-related publications on kidney diseases from 1999 to 2022 showed a rapid year-by-year increasing trend, with a slight decrease in 2015. The most significant increase was observed in the year 2020 and 2021, while the number of literature records steadily increased. The data for 2022 were incomplete at the time of writing this manuscript. The association between the number of publication years and publications was significantly correlated, with an R^2^ coefficient of 0.8235. Correspondingly, the total number of citations for the regained articles was EVs (*n* = 44,578). The annual citations displayed a similar upward trend, increasing steadily from two in 1999 to 9,748 in 2021, which showed a sudden and steep rise from 7,229 in 2020 to 9,748 in 2021, with a correlation coefficient R^2^ = 0.7754.

### Source of EV-related publications (active countries, institutions, and journals)

**FIGURE 3:**
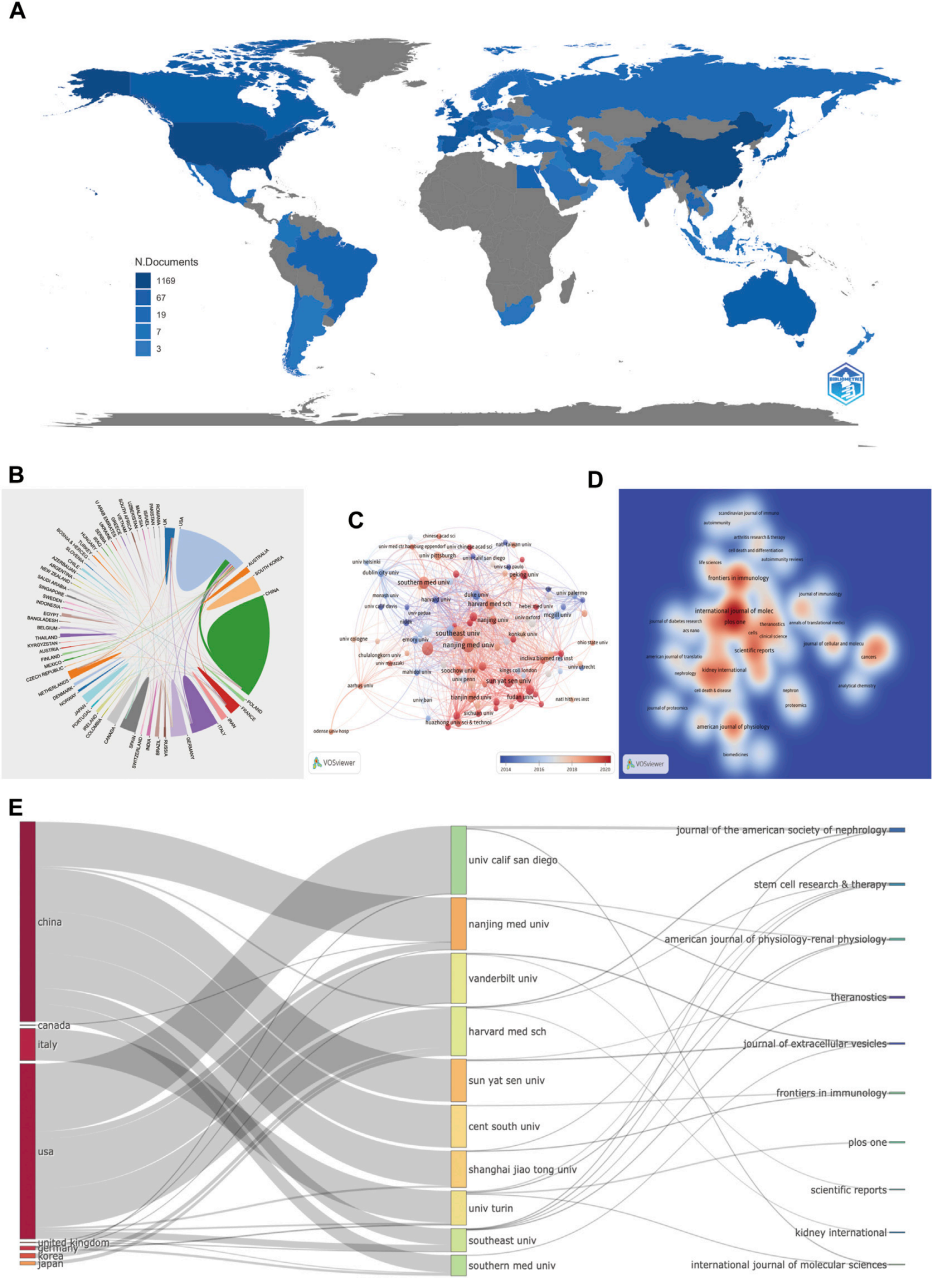
Countries, institutions, and journals related to EVs in kidney disease published worldwide. **(A)** Network map of the country distribution based on R for EVs in kidney disease. **(B)** Contributed countries cooperating based on an online bibliometric platform for EVs in kidney disease. **(C)** The chronological order of institutions produced the articles based on VOSviewer for EVs in kidney disease. **(D)** Journal density map based on VOSviewer for EVs in kidney disease. **(E)** The relationship of the countries, institutions, and journals produced articles based on an alluvial flow map based on R for EVs in kidney disease.

Figure 3 and Tables 1, 2 show that many institutions, countries, and journals contributed to the publications of EVs in kidney-related diseases published worldwide from 1999 to 2022.

**TABLE 1:** Top 10 countries and institutions that contributed to publications of EVs in kidney disease.

Top 10 countries that contributed to publications of EVs in kidney disease							
Rank	Country	Document	Percentage (%)	Citation	Citation/document	Centrality	Total link strength
1	China	351	34.38%	8,317	23.70	0.23	1745
2	United States	280	27.42%	15,654	55.91	0.38	1778
3	Italy	91	8.91%	5,793	63.66	0.2	732
4	Germany	57	5.58%	3,118	54.70	0.19	409
5	Spain	46	4.51%	1,332	28.96	0.02	518
6	England	42	4.11%	2,875	68.45	0.28	326
7	South Korea	40	3.92%	1,102	27.55	0	320
8	France	38	3.72%	3,342	87.95	0.04	207
9	Canada	38	3.72%	2,455	64.61	0.02	224
10	Japan	38	3.72%	1917	50.45	0.01	319
Top 10 institutions that contributed to publications of EVs in kidney disease							
Rank	Institution (Country)	Document	Percentage (%)	Citation	Citation/document	Centrality	Total link strength
1	Southeast University (China)	25	13.97%	8,317	332.68	0.04	242
2	Nanjing Medical University (China)	24	13.41%	15,654	652.25	0.03	99
3	Shanghai Jiao Tong University (China)	22	12.29%	5,793	263.32	0.03	44
4	Southern Medical University (China)	19	10.61%	3,118	164.11	0.04	85
5	Sun Yat-sen University (China)	19	10.61%	1,332	70.11	0.01	101
6	Mayo Clinic (United States)	18	10.06%	2,875	159.72	0.05	186
7	Harvard Medical School (United States)	15	8.38%	1,102	73.47	0.03	111
8	Fudan University (China)	13	7.26%	3,342	257.08	0.01	73
9	Peking University (China)	12	6.70%	2,455	204.58	0.04	34
10	Central South University (China)	12	6.70%	1917	159.75	0	47

**TABLE 2:** Top 10 journals and co-cited journals by papers of extracellular vesicle in kidney disease.

Rank	Journal	Document	Total citation	Citation/document	Total link strength	IF_2021_	H-index	Rank	Co-cited journal	Citations	Total link strength	IF_2021_
1	International Journal of Molecular Sciences	33	290	33.50	172	6.208	9	1	Kidney International	1731	107,964	18.998
2	Frontiers in Immunology	27	648	37.38	111	8.786	12	2	Journal of the American Society of Nephrology	1700	107,929	14.978
3	PLOS ONE	26	1,353	17.80	175	3.752	20	3	PLOS ONE	1,687	137,033	3.752
4	American Journal of Physiology-Renal Physiology	20	944	13.29	134	4.097	13	4	PNAS	1,258	101,300	12.779
5	Scientific Reports	20	568	13.36	60	4.996	11	5	Journal of Biological Chemistry	1,165	90,916	5.486
6	Stem cell Research and Therapy	18	849	29.77	112	8.079	12	6	Journal of Immunology	1,039	91,370	5.426
7	Kidney International	16	2,152	28.54	188	18.998	14	7	Nature	915	75,505	69.504
8	Journal of the American Society of Nephrology	15	1924	24.70	110	14.978	13	8	Scientific Reports	864	71,808	4.996
9	Journal of Extracellular Vesicles	12	411	19.81	50	17.337	8	9	Journal of Extracellular Vesicles	853	68,100	17.337
10	Theranostics	12	411	21.59	41	11.600	10	10	Blood	761	72,377	25.476

PNAS, proceedings of the National Academy of Sciences of the United States.

Among the 63 countries and regions that contributed to the publications in this field, China had the greatest number of publications (351 articles, 34.38% of all the articles), followed by the United States (280, 27.42%), Italy (91, 8.91%), Germany (57, 5.58%), and Spain (46, 4.51%). Comparatively, the United States had the highest number of citations (15,654 citations), followed by China (8,317 citations), Italy (5,793 citations), France (3,342 citations), and Germany (3,118 citations), with the rest having < 3,000 citations. These findings showed that the United States (280 articles, 15,654 citations, 0.38 centralities, and 1778 total link strength) and China (351 articles, 8,317 citations, 0.23 centralities, and 1,745 total link strength) were the two leading countries in the number of citations and links among other countries (Table 1; Figures 3A, B).

It was reported that 1,423 institutions had published articles on EV use in kidney-related diseases in the past 22 years. Among 1,423 institutions that appeared at least five times, the remaining 107 institutions met the thresholds (Figure 3C). The 10 most productive institutions were almost entirely from China, except Harvard Medical School and Mayo Clinic from the United States. The Southeast University (25 articles) was the leading institution in publication, followed by the Nanjing Medical University (24 articles), Shanghai Jiao Tong University (22 articles), Southern Medical University (19 articles), Sun Yat-sen University (19 articles), and Mayo Clinic (18 articles). The Southeast University also showed the most frequent collaboration with other institutions (total link strength = 242 times), which was followed by the Mayo Clinic (186 times), Harvard Medical School (111 times), and Nanjing Medical University (99 times), suggesting their leading role in EVs in kidney-related diseases (Table 1; Figure 3C).

Among 462 productive journals that appeared at least five times, the remaining 48 productive journals met the thresholds (Figure 3D). The 10 most productive journals and co-cited journals on EVs in kidney diseases are displayed in Table 4, which shows that 20.4% of all publications were published in these journals. The top 5 in producing the number of published journals were the International Journal of Molecular Sciences (33 articles), Frontiers in Immunology (27 articles), PLOS ONE (26 articles), American Journal of Physiology-Renal Physiology (20 articles), and Scientific Reports (20 articles). According to the H-index of the top 10 productive journals, PLOS ONE had the largest number of impact measures (20 H-index), followed by Kidney International (14), American Journal of Physiology-Renal Physiology (13), Journal of the American Society of Nephrology (13), and Frontiers in Immunology (12). Interestingly that among these top H-index above were nephrology-related.

Among 4,358 co-cited journals that appeared at least 20 times, the remaining 531 co-cited journals met the thresholds (Figure 3D). Kidney International (1,731 citations and 107,964 total link strength), Journal of the American Society of Nephrology (1,700 citations and 107,929 total link strength), PLOS ONE (1,687 citations and 137,033 total link strength), PNAS (1,258 citations and 101,300 total link strength), and Journal of Biological Chemistry (1,165 citations and 90,916 total link strength) had the most co-citations with other journals.

The relationship between countries, institutions, and journals based on an alluvial flow map or three-field plot (every ten items were set) for EVs in kidney diseases is shown in Figure 3E. China contained or connected with seven targeted institutions (Nanjing Medical University, Sun Yat-sen University, Shanghai Jiao Tong University, Southern Medical University, Central South University, and Harvard Medical School). The United States contained or connected with eight targeted institutions (University of California San Diego, Vanderbilt University, Harvard Medical School, Nanjing Medical University, Southern Medical University, University of Turin, Shanghai Jiao Tong University, and Southern Medical University). The University of California San Diego connected with two targeted journals (Journal of the American Society of Nephrology and International Journal of Molecular Sciences); Nanjing Medical University connected with two targeted journals (American Journal of Physiology-Renal Physiology and Theranostics); Vanderbilt University connected with two targeted journals (Journal of Extracellular Vesicles and Scientific Reports); Harvard Medical School connected with three targeted journals (Kidney International, Journal of the American Society of Nephrology, and Stem Cell Research and Therapy); Sun Yat-sen University connected with two targeted journals (Journal of Extracellular Vesicles and Theranostics); Central South University connected with one targeted journal (Frontiers in Immunology); Shanghai Jiao Tong University connected with two targeted journals (Stem cell Research and Therapy and Frontiers in Immunology); University of Turin connected with four targeted journals (American Journal of Physiology-Renal Physiology, PLOS ONE, Stem Cell Research and Therapy, and International Journal of Molecular Sciences); Southern Medical University connects with four targeted journals (Theranostics, Stem Cell Research and Therapy, American Journal of Physiology-Renal Physiology, and Journal of the American Society of Nephrology); and Southern Medical University connected with one targeted journal (Journal of Extracellular Vesicles). Interestingly, most of the collaborations within the institutions in China or institutions in the United States took place within their respective institutions.

## Analysis of authors, co-authorship, and co-cited references

**FIGURE 4:**
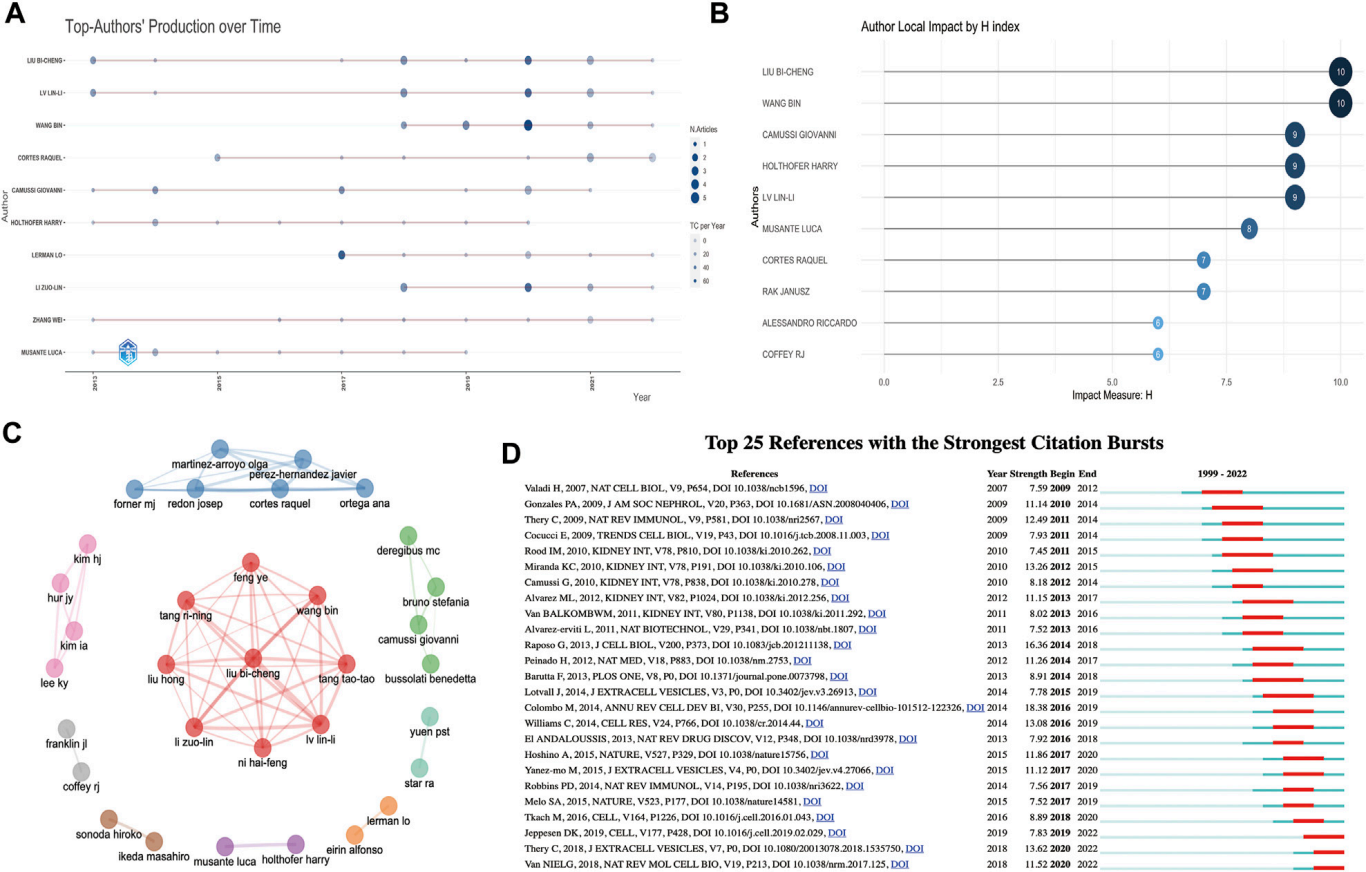
Analysis of authors and references involved in EVs in kidney disease. **(A)** The publication timeline for the 10 most active authors based on R. **(B)** Author local impact by H-index based on R. **(C)** Collaboration network of authors based on R. **(D)** Top 25 references with the strongest citation bursts based on CiteSpace involved in EVs in kidney disease.

The top 10 productive and co-cited authors on EVs in kidney diseases are listed in Table 3. Of the total of 6,486 authors, Liu Bi-cheng (15 articles, 745 citations, and 327 total link strength) and Lv Lin-li (13 articles, 678 citations, and 312 total link strength) were the most prolific authors among all 374 publication documents from all 56 productive authors. In contrast, Thery C (745 citations and 1,398 total link strength) and Bruno S (159 citations and 4,001 total link strength) were the most prolific co-cited authors among all 135,413 citations. Moreover, according to the publication timeline for the 10 most active authors shown in Figure 4A, Liu Bi-cheng, Lin-li Lv, Zhang Wei, Camussi Giovanni, Holthofer Harry, and Cortes Raquel had the longest period in publication on EVs in kidney-related diseases. Liu Bi-cheng and Wang Bing had the highest number of impact measures (H-index, 10), followed by Camussi Giovanni (H-index, 9), Lv Lin-li (H-index, 9), and Holthofer Harry (H-index, 9) (Figure 4B). The collaboration network of authors is shown in Figure 4C. Liu Bi-cheng, Wang Bing, Lv Lin-li, Li Zuo-lin, Tang Tao-tao, Feng Ye, Tang Ri-ning, Liu Hong, and Ni Hai-feng were identified as the biggest collaboration network and active teamwork in this field. Cortes Raquel, Forner MJ, Martinez-arroyo Olga, Ortega Ana, Redon Josep, and Perez-Hernandez Javier were the second biggest collaboration network and active teamwork in this field.

**TABLE 3:** Top 10 authors and co-cited authors in the field of extracellular vesicle in kidney disease.

Rank	Author	Country	Document	Citation	Citation/document	Total link strength	H-index	Rank	Co-cited author	Country	Total citations	Total link strength
1	Liu, Bi-cheng	China	15	745	26.95	327	10	1	Thery, C.	France	283	4,853
2	Lv, Lin-li	China	13	678	24.74	312	9	2	Bruno, S.	Italy	159	4,001
3	Wang, Bin	China	13	512	28.80	268	10	3	Valadi, H.	Sweden	159	2,767
4	Cortes, Raquel	Spain	11	264	22.59	170	7	4	Pisitkun, T.	United States	151	2,462
5	Camussi, Giovanni	Italy	10	672	13.38	91	9	5	Lv, Lin-li	China	146	3,319
6	Li, Zuo-lin	China	9	337	20.70	236	6	6	Zhou, H.	China	141	3,095
7	Lerman, Lilach	United States	9	413	10.10	123	3	7	Raposo, G.	France	140	2,403
8	Holthofer, Harry	Germany	9	293	5.38	74	9	8	Colombo, M.	Italy	111	2,585
9	Zhang, Wei	China	9	279	6.00	47	1	9	Al-nedawi, K.	Canada	93	1,641
10	Tang, Tao-tao	China	8	227	19.04	199	5	10	Mathivanan, S.	Australia	89	1,968

**TABLE 4:** Top 10 co-cited references related to EVs in kidney disease.

Rank	Title	Journal IF_2021_	First author	Publication time	References type	Total citation	Total link strength
1	Exosome-mediated transfer of mRNAs and microRNAs is a novel mechanism of genetic exchange between cells.	Nature Cell Biology (IF = 28.213)	Hadi Valadi	05/2007	Article	158	1,273
2	Identification and proteomic profiling of exosomes in human urine.	PNAS (IF = 12.779)	Trairak Pisitkun	07/2004	Article	123	1,028
3	Extracellular vesicles: exosomes, microvesicles, and friends.	Journal of Cell Biology (IF = 8.077)	Graça Raposo	02/2013	Review	97	643
4	Biogenesis, secretion, and intercellular interactions of exosomes and other extracellular vesicles.	Annual Review of Cell and Developmental Biology (IF = 11.902)	Marina Colombo	08/2014	Review	91	953
5	Membrane vesicles as conveyors of immune responses.	Nature Reviews. Immunology (IF = 108.555)	Clotilde Théry	06/2009	Review	89	695
6	Exosomes: composition, biogenesis, and function	Nature Reviews. Immunology (IF = 108.555)	Clotilde Théry	08/2002	Review	71	475
7	Biological properties of extracellular vesicles and their physiological functions.	Journal of Extracellular Vesicles (IF = 17.337)	María Yáñez-Mó	05/2015	Article	69	640
8	Isolation and characterization of exosomes from cell culture supernatants and biological fluids.	Current Protocols in Cell Biology (IF = 8.386)	Clotilde Théry	04/2006	Article	69	567
9	Nucleic acids within urinary exosomes/microvesicles are potential biomarkers for renal disease.	Kidney International (IF = 18.998)	Kevin C. Miranda	07/2010	Article	64	666
10	Mesenchymal stem cell-derived microvesicles protect against acute tubular injury	Journal of the American Society of Nephrology (IF = 14.978)	Stefania Bruno	04//2009	Article	62	698

PNAS, proceedings of the National Academy of Sciences of the United States.

The top 10 co-cited references related to EVs in kidney-related diseases are listed in Table 4. Of the 45,959 cited references, 130 co-cited references were cited at least 20 times in EVs (Supplementary Table S2). Valadi et al. (2007) (2007, 158 citations, and 1,273 total link strength), Pisitkun et al. (2004) (2004, 123 citations, and 1,028 total link strength), and Raposo and Stoorvogel (2013) (2013, 97 citations, and 643 total link strength) were the most selected prolific co-cited references among all 45,959 cited references. We also established or extracted highly influential or major achievement articles (Table 5) *via* co-cited references based on VOSviewer, Citespace, and the R bibliometrics package (Supplementary Tables S2, S4). Interestingly, most of the top 10 co-cited references and the top 8 publications were focused on basic biogenesis, secretion, function, identification, and proteomic profiling of exosomes or EVs. Four publications were reviews, and four others were articles, except two articles reported by Miranda et al. (2010), which concentrated on urinary exosomes as a novel and non-invasive source of nucleic acids for further investigation of renal disease biomarkers to find new potential treatments. In a publication by Bruno et al. (2009), the authors focused on AKI recovery and reported that AKI might be improved using microvesicles derived from mesenchymal stem cells (MSCs) by disrupting the vertical transfer of mRNA from mesenchymal stem cells to surviving tubular cells.

**TABLE 5:** Major achievement articles on EVs in kidney-related diseases.

Title	First author	Journal	Year	Main conclusion	References
Identification and proteomic profiling of exosomes in human urine	Pisitkun T.	PNAS	2004	They reported that exosome isolation may provide an efficient first step in biomarker discovery in urine.	Pisitkun et al. (2004)
Exosomal Fetuin-A identified by proteomics: a novel urinary biomarker for detecting acute kidney injury.	Zhou H.	Kidney International	2006	They concluded that (1) proteomic analysis of urinary exosomes can provide biomarker candidates for the diagnosis of AKI and (2) urinary Fetuin-A might be a predictive biomarker of structural renal injury.	Zhou et al. (2006b)
Urinary exosomal transcription factors, a new class of biomarkers for renal disease	Zhou H.	Kidney International	2008	They suggested that transcription factor ATF3 may provide a novel renal tubular cell biomarker for acute kidney injury, while WT-1 may detect early podocyte injury. Measurement of urinary exosomal transcription factors may offer insight into cellular regulatory pathways.	Zhou et al. (2008)
Large-scale proteomics and phosphoproteomics of urinary exosomes.	Gonzales P.	Journal of the American Society of Nephrology	2009	They demonstrated the potential use of exosome analysis to identify a genetic renal disease.	Gonzales et al. (2009)
Nucleic acids within urinary exosomes/microvesicles are potential biomarkers for renal disease	Miranda K.	Kidney International	2010	They showed the routine isolation and use of urinary microvesicles as a novel and non-invasive source of nucleic acids to further renal disease biomarker discovery.	Miranda et al. (2010)
Microvesicles derived from human adult mesenchymal stem cells protect against ischaemia-reperfusion-induced acute and chronic kidney injury	Gatti S.	Nephrology Dialysis Transplantation	2011	MVs released from MSCs protect from AKI induced by ischemia-reperfusion injury and from subsequent chronic renal damage. This suggests that MVs could be exploited as a potential new therapeutic approach.	Gatti et al. (2011)
Proteomic analysis of urinary exosomes from patients of early IgA nephropathy and thin basement membrane nephropathy	Moon P.	Proteomics	2011	They showed the possibility of identifying biomarker candidates for human urinary diseases using urinary exosomes and might help to understand the pathophysiology of early IgAN and TBMN at the protein level.	Moon et al. (2011)
Microvesicles derived from mesenchymal stem cells enhance survival in a lethal model of acute kidney injury	Bruno S.	PLOS ONE	2012	They found that microvesicles (MVs) released from MSCs were found to exert a pro-survival effect on renal cells *in vitro* and *in vivo*, suggesting that MVs may contribute to renal protection conferred by MSCs.	Bruno et al. (2012)
Comparison of protein, microRNA, and mRNA yields using different methods of urinary exosome isolation for the discovery of kidney disease biomarkers	Alvarez M.	Kidney International	2012	They revealed a simple, fast, highly scalable, and effective alternative for the isolation of exosomes that may facilitate the identification of exosomal biomarkers from urine.	Alvarez et al. (2012)
Urinary exosomal microRNAs in incipient diabetic nephropathy.	Barutta F.	PLOS ONE	2013	They found that urinary exosomal miRNA content is altered in type 1 diabetic patients with incipient diabetic nephropathy and that miR-145 may represent a novel candidate biomarker/player in the complication.	Barutta et al. (2013)
MicroRNA-29c in urinary exosome/microvesicle as a biomarker of renal fibrosis.	Lv L.	American Journal of Physiology-Renal Physiology	2013	They found that miR-29c in urinary exosome correlates with both renal function and degree of histological fibrosis, suggesting it as a novel, non-invasive marker for renal fibrosis.	Lv et al. (2013)
Diabetic nephropathy induces changes in the proteome of human urinary exosomes as revealed by label-free comparative analysis.	Zubiri I.	Journal of Proteomics	2014	They declared that a panel of three proteins (AMBP, MLL3, and VDAC1) responding to diabetic nephropathy was discovered. Diabetic nephropathy induces changes in the proteome of human urinary exosomes as revealed by label-free comparative analysis.	Zubiri et al. (2014)
Characterization and deep sequencing analysis of exosomal and non-exosomal miRNA in human urine.	Cheng L.	Kidney International	2014	Their study extensively characterizes the RNA content of exosomes isolated from urine, providing the potential to identify miRNA biomarkers in human urine.	Cheng et al. (2014)
Subfractionation, characterization, and in-depth proteomic analysis of glomerular membrane vesicles in human urine.	Hogan M.	Kidney International	2014	They revealed sample sizes required to identify new glomerular disease biomarkers, expand the ELV proteome, and provide a reference proteome in a database that may prove useful in the search for biomarkers of glomerular disease.	Hogan et al. (2014)
miR-29c in urinary exosomes as predictor of early renal fibrosis in lupus nephritis.	Solé C.	Nephrol Dialysis Transplantation	2015	They suggested that miR-29c could be used as a novel non-invasive marker of early progression to fibrosis in patients with lupus nephritis.	Solé et al. (2015)
AKI recovery induced by mesenchymal stromal cell-derived extracellular vesicles carrying microRNAs.	Collino F.	Journal of the American Society of Nephrology	2015	They proclaimed that AKI recovery was induced by mesenchymal stromal cell-derived extracellular vesicles carrying microRNAs.	Collino et al. (2015)
Mesenchymal stem cells deliver exogenous microRNA-let7c *via* exosomes to attenuate renal fibrosis.	Wang B.	Molecular Therapy	2016	They proved that the effective antifibrotic function of engineered MSCs is able to selectively transfer miR-let7c to damaged kidney cells and will pave the way for the use of MSCs for therapeutic delivery of miRNA targeted at kidney disease.	Wang et al. (2016)
Exosomes secreted by human urine-derived stem cells could prevent kidney complications from type I diabetes in rats.	Jiang Z.	Stem Cell Research and Therapy	2016	They concluded that exosomes from conditioned medium of urine-derived stem cells (USCs-Exo) may have the potential to prevent kidney injury from diabetes by inhibiting podocyte apoptosis and promoting vascular regeneration and cell survival.	Jiang et al. (2016)
Transfer of microRNA-486-5p from human endothelial colony forming cell-derived exosomes reduces ischemic kidney injury.	Viñas J.	Kidney International	2016	They determined that delivery of ECFC exosomes reduces ischemic kidney injury *via* transfer of miR-486-5p targeting PTEN. Exosomes enriched in miR-486-5p could represent a therapeutic tool in acute kidney injury.	Viñas et al. (2016)
Mesenchymal stromal cells-derived extracellular vesicles ameliorate acute renal ischemia-reperfusion injury by inhibition of mitochondrial fission through miR-30	Gu D.	Stem Cells International	2016	They reported that the single administration of human Wharton Jelly mesenchymal stromal cells-extracellular vesicles (hWJMSC-EVs) could protect the kidney from IRI by inhibition of mitochondrial fission *via* miR-30.	Gu et al. (2016)
Combination of adipose-derived mesenchymal stem cells (ADMSC) and ADMSC-derived exosomes for protecting the kidney from acute ischemia-reperfusion injury.	Lin K.	International Journal of Cardiology	2016	They showed that combined exosome-adipose-derived mesenchymal stem cell (ADMSC) therapy was superior to either one for protecting kidneys from acute ischemia-reperfusion injury.	Lin et al. (2016)
Urinary exosomal miRNA signature in type II diabetic nephropathy patients.	Delić D.	PLOS ONE	2016	They indicated that urinary exosomal miRNA content is altered in type II diabetic patients with diabetic nephropathy (DN). Deregulated miR-320c, which might have an impact on the TGF-β-signaling pathway *via* targeting thrombospondin 1 (TSP-1), shows promise as a novel candidate marker for disease progression in type II DN.	Delić et al. (2016)
The effects of glomerular and tubular renal progenitors and derived extracellular vesicles on recovery from acute kidney injury.	Ranghino A.	Stem Cell Research and Therapy	2017	They demonstrated that glomeruli-mesenchymal stromal cells (Gl-MSCs) may contribute to the recovery of mice with AKI induced by ischemia-reperfusion injury primarily through the release of EV	Ranghino et al. (2017)
Mesenchymal stem cell-derived extracellular vesicles attenuate kidney inflammation.	Eirin A.	Kidney International	2017	They declared that renoprotective benefits were attenuated in IL10-depleted pigs. Extracellular vesicle-based regeneration techniques may be effective for individuals with metabolic syndrome and renal artery stenosis.	Eirin et al. (2017)
Renal tubular cell-derived extracellular vesicles accelerate the recovery of established renal ischemia-reperfusion injury.	Dominguez J.	Journal of the American Society of Nephrology	2017	They found that EV treatment significantly improved renal tubular damage, 4-hydroxynanoneal adduct formation, neutrophil infiltration, fibrosis, and microvascular pruning. EV therapy also markedly reduced the large renal transcriptome drift observed after ischemia and the potential utility of EV to limit severe renal ischemic injury after the occurrence.	Dominguez et al. (2017)
Exosomal CCL2 from tubular epithelial cells is critical for albumin-induced tubulointerstitial inflammation	Lv L.	Journal of the American Society of Nephrology	2018	They demonstrated that the increasing release of exosomes that transfer CCL2 mRNA from TECs to macrophages constitutes a critical mechanism of albumin-induced tubulointerstitial inflammation associated with CKD.	Lv et al. (2018b)
Mesenchymal stem cell-derived exosomes ameliorated diabetic nephropathy by autophagy induction through the mTOR signaling pathway.	Ebrahim N.	Cells	2018	They concluded that autophagy induction by exosomes could attenuate DN in a rat model of streptozotocin-induced diabetes mellitus.	Ebrahim et al. (2018)
Identification of urinary exosomal non-coding RNAs as novel biomarkers in chronic kidney disease	Khurana R.	RNA	2018	They confirmed that miRNA-181a appeared as the most robust and stable potential biomarker, being significantly decreased by about 200-fold in exosomes of CKD patients compared to healthy controls. Using a cell culture system for CKD indicated that urinary exosomes might indeed originate from renal proximal tubular epithelial cells.	Khurana et al. (2017)
HIF-1α inducing exosomal microRNA-23a expression mediates the cross-talk between tubular epithelial cells and macrophages in tubulointerstitial inflammation.	Li Z.	Kidney International	2019	They showed that blockade of exosome-mediated miRNA-23a transfer between tubular epithelial cells and macrophages may serve as a novel therapeutic approach to ameliorate tubulointerstitial inflammation in the kidney.	Li et al. (2019)
Employing macrophage-derived microvesicle for kidney-targeted delivery of dexamethasone: An efficient therapeutic strategy against renal inflammation and fibrosis.	Tang T.	Theranostics	2019	They showed that macrophage-derived MVs efficiently deliver DEX into the inflamed kidney and exhibit a superior capacity to suppress renal inflammation and fibrosis without apparent glucocorticoid adverse effects. Their findings demonstrate the effectiveness and security of a novel drug delivery strategy with promising clinical applications.	Tang et al. (2019c)
Exosome secreted from adipose-derived stem cells attenuates diabetic nephropathy by promoting autophagy flux and inhibiting apoptosis in podocyte.	Jin J.	Stem Cell Research and Therapy	2019	They illustrated that adipose-derived stem cell-derived exosome (ADSC-Exo) vividly ameliorated DN symptoms by enhancing the expression of miR-486, which led to the inhibition of the Smad1/mTOR signaling pathway in podocyte. Possibly, ADSC-Exo can be used as a main therapeutic strategy for DN in the future.	Jin et al. (2019)
Exosome-mediated miR-29 transfer reduces muscle atrophy and kidney fibrosis in mice.	Wang H.	Molecular Therapy	2019	They concluded that Exo/miR29 ameliorates skeletal muscle atrophy and attenuates kidney fibrosis by downregulating YY1 and TGF-β pathway proteins.	Wang et al. (2019b)
Exosomes derived from GDNF-modified human adipose mesenchymal stem cells ameliorate peritubular capillary loss in tubulointerstitial fibrosis by activating the SIRT1/eNOS signaling pathway.	Chen L.	Theranostics	2020	They reported that exosomes derived from GDNF-modified human adipose mesenchymal stem cells ameliorate peritubular capillary loss in tubulointerstitial fibrosis by activating the SIRT1/eNOS signaling pathway.	Chen et al. (2020)
*In Vivo* tracking of mesenchymal stem cell-derived extracellular vesicles improving mitochondrial function in renal ischemia-reperfusion injury.	Cao H.	ACS Nano	2020	They concluded that MSC-EVs accumulated in the renal tubules during renal I/R injury and promoted the recovery of kidney function *via* activating the Keap1-Nrf2 signaling pathway and enhancing mitochondrial function of TECs. DPA-SCP with AIE characteristics allows non-invasive and precise *in vivo* visualization of MSC-EVs in kidney repair.	Cao et al. (2020a)
Exosomes derived from hucMSC attenuate renal fibrosis through CK1δ/β-TRCP-mediated YAP degradation.	Ji C.	Cell Death and Disease	2020	They suggested that hucMSC-Ex attenuates renal fibrosis through CK1δ/β-TRCP inhibited YAP activity, unveiling a new mechanism for the therapeutic effects of hucMSC-Ex on tissue injury and offering a potential approach for renal fibrosis treatment.	Ji et al. (2020)
Enhanced therapeutic effects of MSC-derived extracellular vesicles with an injectable collagen matrix for experimental acute kidney injury treatment.	Liu Y.	Stem Cell Research and Therapy	2020	They indicated the collagen matrix markedly enhanced the retention of EVs and further augmented the therapeutic effects of EVs for AKI. This strategy for improving the efficacy of EV therapy provides a new direction for cell-free therapy.	Liu et al. (2020a)
Supramolecular nanofibers containing arginine–glycine–aspartate (RGD) peptides boost therapeutic efficacy of extracellular vesicles in kidney repair.	Zhang C.	ACS Nano	2020	They illustrated that (Arg-Gly-Asp) RGD hydrogels facilitated MSC-derived let-7a-5p-containing EVs, improving reparative potential against AKI. This study developed an RGD scaffold to increase the EV integrin-mediated loading and, in turn, improved therapeutic efficacy in renal repair; therefore, this strategy shed light on MSC-EV application as a cell-free treatment for potentiated efficiency.	Zhang et al. (2020)
Extracellular vesicle-encapsulated IL-10 as novel nanotherapeutics against ischemic AKI.	Tang T.	Science Advances	2020	They demonstrated that EVs can serve as a promising delivery platform to manipulate IL-10 for the effective treatment of ischemic AKI.	Tang et al. (2020a)
Urinary extracellular vesicles carrying klotho improve the recovery of renal function in an acute tubular injury model.	Grange C.	Molecular Therapy	2020	They revealed a novel potential use of urinary extracellular vesicles (uEVs) as a new therapeutic strategy for acute kidney injury, highlighting the presence and role of the renoprotective factor Klotho.	Grange et al. (2020)
Three-dimensional culture of MSCs produces exosomes with improved yield and enhanced therapeutic efficacy for cisplatin-induced acute kidney injury.	Cao J.	Stem Cell Research and Therapy	2020	They demonstrated that the hollow fiber 3D culture system provides an efficient strategy for the continuous production of MSC-exos, which has enhanced therapeutic potential for cisplatin-induced AKI.	Cao et al. (2020b)
Tubule-derived exosomes play a central role in fibroblast activation and kidney fibrosis	Liu X.	Kidney International	2020	Their results suggested that tubule-derived exosomes play an essential role in renal fibrogenesis through shuttling the Shh ligand. Hence, strategies targeting exosomes could be a new avenue in developing therapeutics against renal fibrosis.	Liu et al. (2020b)
Exosomal miR-125b-5p deriving from mesenchymal stem cells promotes tubular repair by suppression of p53 in ischemic acute kidney injury.	Cao J.	Theranostics	2021	They provided a novel insight into the role of MSC-exos in renal tubule repair and highlight the potential of MSC-exos as a promising therapeutic strategy for AKI.	Cao et al. (2021b)
Exosomes derived from BM-MSCs mitigate the development of chronic kidney damage post-menopause *via* interfering with fibrosis and apoptosis.	Alasmari W.	Biomolecule	2022	They revealed that exosomes derived from bone marrow mesenchymal stem/stromal cells (BM-MSCs) mitigate the development of chronic kidney damage post-menopause *via* interfering with fibrosis and apoptosis.	Alasmari et al. (2022)

PNAS, proceedings of the National Academy of Sciences of the United States.

Additionally, “bursts” refer to references appearing over a period of time and reflect the popular topics during that period. The top 25 co-cited references with the strongest citation bursts are shown in Figure 4D. The findings indicated that the first instance of a citation burst occurred in 2009, and the most recent instance of a reference with a citation burst was recorded in 2020. The highest burst strength for EVs in kidney-related diseases was from Colombo et al. (2014) (18.38 strength), Raposo and Stoorvogel (2013) (16.36 strength), and Théry et al. (2018) (13.62 strength). Moreover, Tkach and Théry (2016), Jeppesen et al. (2019), Thery et al. (2018), and van Niel et al. (2018) received more attention in recent years. Interestingly, among the top 25 co-cited references, five co-cited references (20%) were from Kidney International, among which three were in the 2010 year strength (Camussi et al., 2010; Miranda et al., 2010; Rood et al., 2010), one was in 2011 (van Balkom et al., 2011), and one was in 2012 (Alvarez et al., 2012) (Figure 4D).

### Analysis of keywords

**FIGURE 5:**
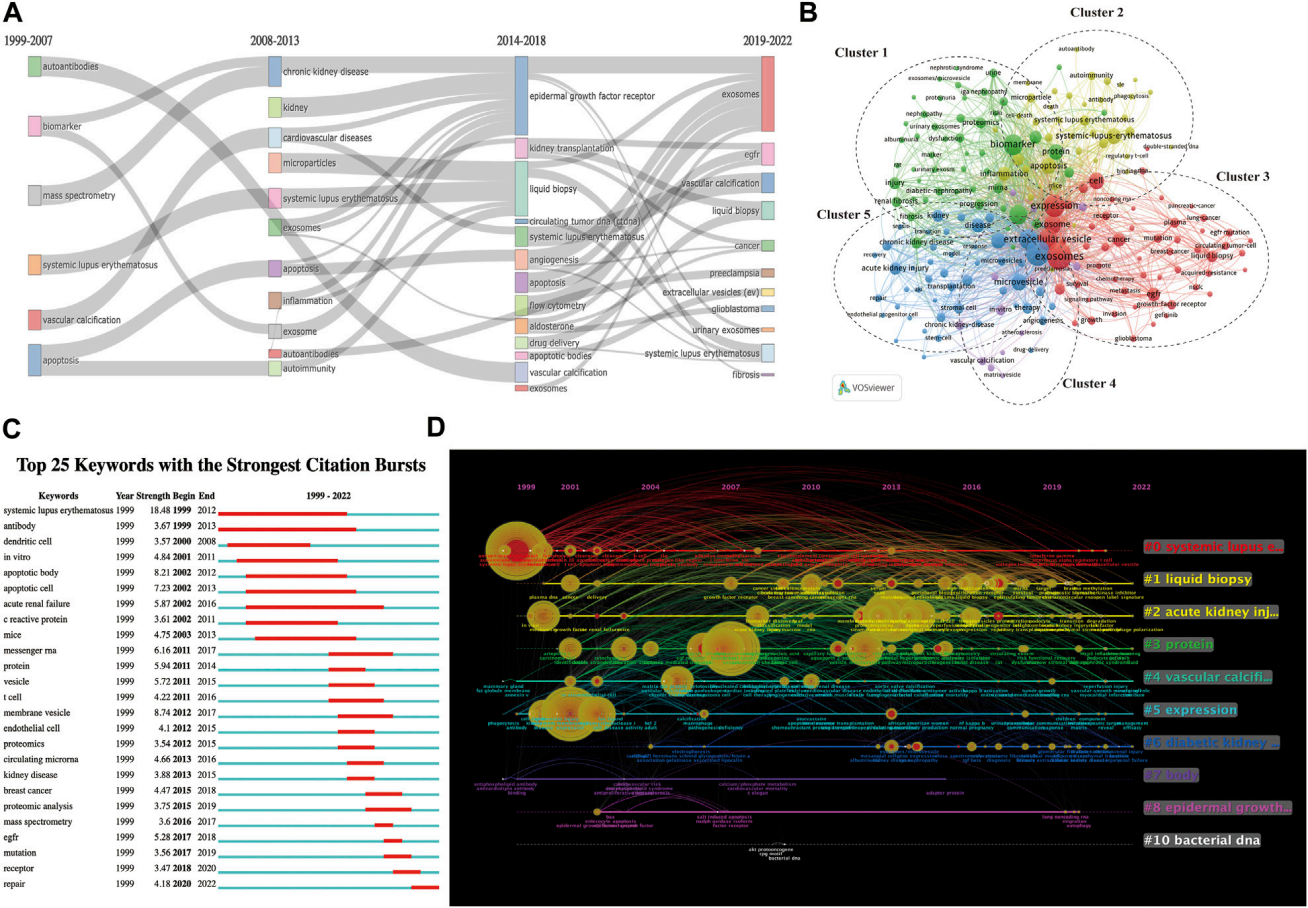
Keyword-related mapping in studies on EVs in kidney disease. **(A)** Major keywords evolution based on R for EVs in kidney disease research. **(B)** Visualization based on keyword co-occurrence relationship based on VOSviewer for EVs in kidney disease. In this network map, keywords with close relationships are assigned to one cluster with the same color. All the keywords could be divided into five clusters: cluster 1 (green nodes), cluster 2 (yellow nodes), cluster 3 (red nodes), cluster 4 (purple nodes), and cluster 5 (blue nodes). **(C)** Top 25 keywords with the strongest bursts by CiteSpace. **(D)** The timeline view of keywords based on CiteSpace related to EVs in kidney disease.

The early stage (1999–2007) of inquiry on EVs in kidney-related disorders was mostly focused on “autoantibodies,” “biomarkers,” “systemic lupus erythematosus,” and “apoptosis,” as shown by the thematic evolution analysis of the author’s keywords based on publication year. These findings indicate that knowledge of EVs and kidney-related diseases was not well-understood in this stage, except for “systemic lupus erythematosus.” In the platform stage, the critical challenge of kidney-related diseases has steadily developed toward “chronic renal disease” and “kidney,” a breakthrough from 2008 to 2013. This occurred between the years 2008 and 2013. However, between the years 2014 and 2018, a shift was seen toward the concepts of “kidney transplantation,” “liquid biopsy,” “acute kidney injury,” “diabetic nephropathy,” “epigenetics,” and “urinary exosomes.” The terms “liquid biopsy” and “urinary exosomes” have captured the interest of academics throughout the course of the last three years (Figure 5A; Supplementary Table S3).

The top 25 co-occurrence keywords related to EVs in kidney-related diseases are shown in Table 6, suggesting that exosomes were the most frequent keywords (264 occurrences and 622 total link strength), followed by exosm (253 occurrences and 696 total link strength) and expression (193 occurrences and 530 total link strength). Among the top 25 keywords, it was seen that biomarker (167 occurrences and 501 total link strength), microRNA (138 occurrences and 415 total link strength), systemic lupus erythematosus (92 occurrences and 140 total link strength), EGFR (72 occurrences and 190 total link strength), acute kidney injury (62 occurrences and 171 total link strength), kidney (54 occurrences and 168 total link strength), liquid biopsy (53 occurrences and 139 total link strength), and chronic kidney disease (53 occurrences and 141 total link strength) were the most mentioned related to kidney diseases.

**TABLE 6:** Top 25 co-occurrence keywords related to EVs in kidney disease.

Rank	Keyword	Occurrences	Total link strength
1	Exosomes	264	622
2	Exosm	253	696
3	Expression	193	530
4	Extracellular vesicle	168	435
5	Biomarker	167	501
6	Extracellular vesicles	148	441
7	MicroRNA	138	415
8	Exosome	131	310
9	Microvesicle	109	354
10	Cell	106	306
11	Apoptosis	101	187
12	Mechanism	95	293
13	Protein	93	289
14	Systemic lupus erythematosus	92	140
15	Disease	77	216
16	Inflammation	75	193
17	Cancer	73	217
18	EGFR	72	190
19	Identification	65	220
20	Acute kidney injury	62	171
21	Activation	56	140
22	Injury	55	156
23	Kidney	54	168
24	Liquid biopsy	53	139
25	Chronic kidney disease	53	141

A total of 4,520 keywords were obtained, among which 424 keywords appeared at least 10 times (Figure 5B). As shown in Figure 5B, all of the keywords could be divided into the following five groups: cluster 1 (green nodes focus on the use of EVs as biomarkers for potential treatments or clinical applications in kidney-related diseases, including diabetic nephropathy, IgA nephropathy, nephrotic syndrome, and renal fibrosis), cluster 2 (yellow nodes focus on the use or functions of EVs as potential treatments of kidney-related diseases, including systemic lupus erythematosus and lupus nephritis), cluster 3 (red nodes focus on the mechanism of EVs in others diseases, including lung cancer and pancreatic cancer), cluster 4 (purple nodes focus on EV-related material, including drug delivery and serum), and cluster 5 (blue nodes focus on the mechanism or clinical applications from EVs in kidney-related diseases, including CKD and AKI).

The top 25 co-cited keywords with the strongest citation bursts based on Citespace are shown in Figure 5C. The highest burst strength relevant to kidney-related diseases was from systemic lupus erythematosus (1999), acute renal failure (2002), and kidney diseases (2013). Moreover, EGFR (2017), mutation (2017), receptor (2018), and repair (2020) received more attention in recent years (Figure 5C).

We applied the CiteSpace software to cluster the keywords and references to illustrate a timeline for keywords after clustering (Figure 5D; Supplementary Table S4). A total 10 clusters were formed: 1) systemic lupus erythematosus, 2) liquid biopsy, 3) acute kidney injury, 4) protein, 5) vascular calcification, 6) expression, 7) diabetic kidney disease, 8) body, 9) epidermal growth factor, and 10) bacterial DNA. According to the timeline view (Figure 5D), we found that the 1) systemic lupus erythematosus, 2) liquid biopsy, 3) acute kidney injury, 4) protein, 5) vascular calcification, 6) expression, and 7) diabetic kidney disease appeared to be significant and proceeded to develop between EVs and kidney diseases. 1) systemic lupus erythematosus, 2) liquid biopsy, 3) acute kidney injury, and 4) protein had the most prolonged period of attention and effects on EVs in kidney-related diseases.

**FIGURE 6:**
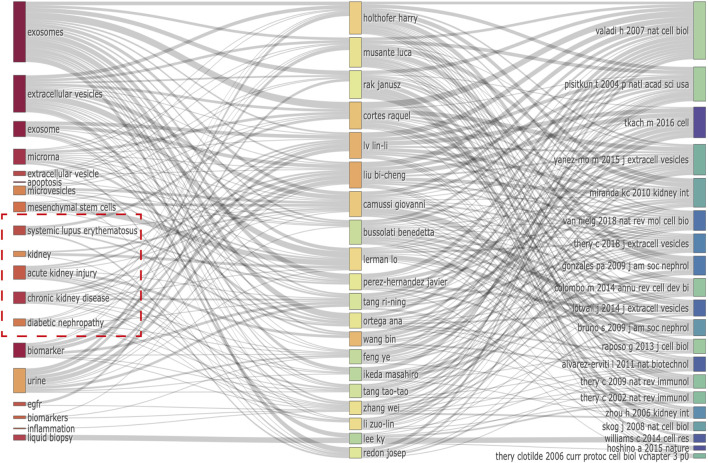
Relationship of the top 20 co-cited references, authors, and keywords evolution based on an alluvial flow map by R.

Notably, we discovered numerous potentially unique characteristics of EVs in kidney-related diseases. Notably, we identified important keywords from relationships between the top 20 co-cited references, authors, and keywords evolution of EVs in kidney-related diseases (Figure 6), including “systemic lupus erythematosus,” “kidney,” “acute kidney injury,” “chronic kidney disease,” and “diabetic nephropathy.” They were almost connected with the top 10 authors and co-cited references (Tables 3, 4).

## Discussion

Based on visual management software, we conducted a comprehensive bibliometric analysis of literature published on EVs and kidney-related diseases from the inception to 26 June 2020, most of which were published after 1999. A total of 1,122 EVs and kidney-related disease publications were obtained from the WoSCC. The number of EVs in kidney disease studies started in 2019 was >100. The interesting integrated evolution in the year 2019 demonstrated the forcefully increasing attention in the international arena on EVs for dealing with kidney-related diseases. A few recent bibliometric studies of EVs regarding improving the field of neuroscience (Long et al., 2022), mesenchymal stem cell-derived extracellular vesicles (MSC-EVs) (Zhang et al., 2022), liver diseases (Shao et al., 2022), and breast cancer (Teles et al., 2021) found a major evolution trend in annual publications around 2012–2016, suggesting that there have been rapid developments on EVs in the last decade.

According to the results in Table 1 and Figures 3A, B, China and the United States were the top 2 countries in publishing literature and linking to other countries. Comparatively, China was the top country in publishing literature and an active country in recent years. Conversely, the United States was the top country in the number of citations and total link strengths in this field. Moreover, China’s centrality and total link strengths were 0.23 and 1,745, while that of the United States were 0.38 and 1,778, respectively. In addition, of the top 10 institutions with the most studied items, eight were from China and two were from the United States. However, the top centrality and total link strength of Chinese institutions were 0.04 and 242 from the Southeast University, respectively, while those of the United States institutions were 0.05 and 186 from the Mayo Clinic, respectively. Therefore, the United States, China, and their corresponding institutions retained the leading status and engagement on EVs in kidney-related disease research, followed by Italy, Germany, Spain, and England, which had high centrality and total link strength, implying that they played important roles in the worldwide collaboration of EVs in kidney-related diseases research. Overall, the publication and citation number outcomes demonstrated that studies related to EVs in kidney-related diseases are still in a high-speed evolution stage, causing significant interest for future research.

Based on the number of citations and articles, total link strength, and H-index, PLOS ONE, Kidney International, American Journal of Physiology-Renal Physiology, Journal of the American Society of Nephrology, Frontiers in Immunology, Stem Cell Research and Therapy, International Journal of Molecular Sciences, and Theranostics were the most influential journals for researchers in EV-related kidney diseases. The most co-cited journals were also from Kidney International, the Journal of the American Society of Nephrology, and PLOS ONE (Table 2). Overall, these results also insinuate that journals in nephrology and on EVs might have a certain influence over a central role and balance on the publication of this study, suggesting the hotness in publishing high-impact papers in this field (Table 2).

Our results on authors’ analysis (Table 3; Figures 4A–C) showed that Liu Bi-cheng (corresponding author) from the Institute of Nephrology, Southeast University School of Medicine, was the most prolific author. He mainly focused on the association of EVs with kidney-related diseases (AKI, CKD, renal inflammation and fibrosis, diabetic nephropathy, etc.). Moreover, he and his colleagues (Lv Lin-li, top 2; Wang Bin, top 3; Tang Tao-tao, top 10; Li Zuo-lin, top 9) also contributed to the correlation between EV and kidney-related disease research (Lv et al., 2018a; Tang et al., 2019a; Wang et al., 2019a; Tang et al., 2019b; Lv et al., 2019; Tang and Liu, 2019; Tang et al., 2020a; Tang et al., 2020c; Wang et al., 2020; Cao et al., 2021a; Feng et al., 2021a; Feng et al., 2021b; Tang et al., 2021; Ding et al., 2022; Tang et al., 2022). Interestingly, these researchers not only had the biggest collaboration network and active teamwork in this field but also the longest period in publication and the highest number of impact measures. Camussi Giovanni (corresponding author) from the Department of Medical Sciences, University of Turin, was the top 5 prolific author for EVs in kidney disease. He mainly focused on the association of EVs with kidney-related diseases (AKI, CKD, systemic lupus erythematosus, ANCA-vasculitis, kidney fibrosis, and diabetic nephropathy) (Biancone and Camussi, 2014; Collino et al., 2015; Lener et al., 2015; Bussolati and Camussi, 2017; Dimuccio et al., 2020; Grange et al., 2020; Kholia et al., 2020; Ramírez-Bajo et al., 2020; Tetta et al., 2020; Kholia et al., 2021; Mazzariol et al., 2021; Bruno et al., 2022; Franzin et al., 2022). Additionally, Cortes Raquel (corresponding author) from the Cardiometabolic and Renal Risk Research Group, INCLIVA Biomedical Research Institute, was the top 4 prolific author for EVs in kidney-related diseases. Notably, he and his team mainly focused on the association of EVs with kidney-related diseases (CKD, kidney-related cardiovascular risk, hypertension, diabetes mellitus, lupus nephritis, and systemic lupus erythematosus) (Perez-Hernandez and Cortes, 2015; Perez-Hernandez et al., 2015; Perez-Hernandez et al., 2016; Perez-Hernandez et al., 2017; Perez-Hernandez et al., 2018a; Perez-Hernandez et al., 2018b; Olivares et al., 2018; Olivares et al., 2020; Ortega et al., 2020; Perez-Hernandez et al., 2021a; Perez-Hernandez et al., 2021b; Redon et al., 2021; Martinez-Arroyo et al., 2022). Overall, research on EVs in kidney-related diseases showed a good link for co-partnership among authors. Furthermore, most scholars were from different countries, and the cooperation was mostly confined to the research team. Researchers from different countries should reinforce collaboration and partake in beneficial terraces to achieve more significant progress and improve the clinical translation of research and the exchange of technological innovation among scientists working on different aspects between EVs and kidney-related diseases.

As shown in Table 4, of the 45,959 cited references, 130 co-cited references were cited at least 20 times in EVs (Table 4; Supplementary Table S2). We found that the co-cited references reported by Valadi et al. (2007) from Nature Cell Biology (IF = 28.213) titled “Exosome-mediated transfer of mRNAs and microRNAs are a novel mechanism of genetic exchange between cells” was the top in the number of citations and total link strengths in EVs in kidney-related diseases. All of them mainly focused on the significant use, formation, targeting, and function of EVs in kidney-related diseases, which included cancer and other diseases (Théry et al., 2002; Pisitkun et al., 2004; Théry et al., 2006; Valadi et al., 2007; Bruno et al., 2009; Théry et al., 2009; Miranda et al., 2010; Colombo et al., 2014; Yáñez-Mó et al., 2015). Additionally, Figure 4D and Table 5 results indicate that the most influential papers were published in basic and clinical journals. As a relatively rapid translation of basic research to clinical research on EVs and kidney-related diseases, the research on these topics forms a good connection between basic and clinical studies, suggesting a favorable development pattern on EVs in kidney disease research.

Keywords are the investigation focus and core objects of a study. Moreover the analysis of keywords suggested that the significant benefit of EVs in kidney-related diseases was research hotspots and keyword co-occurrence analysis. According to the keywords reported, the major keywords included evolution, network map, and top 25 co-occurrence keywords of EVs in kidney disease (Figures 5A, B; Table 6). The top 25 keywords with the strongest bursts and the timeline view of keywords related to EVs in kidney disease are shown in Figures 5C, D. It is possible to realize the allocation and growth of distinct research hotspots in this field. Subsequently, based on the results in the timeline viewer analysis of clustering, we defined the research hotspots and growth frontiers in the field of EVs in kidney-related diseases.

All 13 largest clusters are the results conducted by Citespace and those results were input intoSupplementary Table S4. This review described that urinary extracellular vesicles might have potential pathophysiologic, diagnostic, and therapeutic roles in renal diseases (Erdbrügger and Le, 2016). Cluster #1 was “acute kidney injury, extracellular vesicle, and stem cell-derived”. A review by Jin et al. (2021) entitled “Exosomes: Emerging therapy delivery tools and biomarkers for kidney diseases” mainly focused on the application of nanometer-sized small EVs coated with bilayer structure in diagnosis and their positive effects on the repair of kidney dysfunction and the designated mechanisms. Cluster #6 was “mesenchymal stem, extracellular vesicle, and stem cell-derived” and showed that mesenchymal stem cells-derived EVs could be a promising concept for the repair of damaged kidneys (Nargesi et al., 2017). Cluster #7 was “systemic lupus erythematosus and exosome-based drug delivery system.” This review focused on the crucial traits of nanometer-sized lipid-bilayer-enclosed EVs or exosome-based drug delivery systems in systemic lupus erythematosus (SLE) (Ortega et al., 2020). Cluster #13 was “human islet, extracellular vesicle, and extracellular vesicle.” This review focused on therapeutic options for autoimmune diseases (rheumatoid arthritis, autoimmune type 1 diabetes mellitus, and systemic lupus erythematosus) based on MSCs and MSC-EVs (Martinez-Arroyo et al., 2022). Cluster #18 was “novel biomarker source, peritoneal dialysis, and extracellular vesicle.” The review reported by Brahmadhi et al. entitled “Exosomal proteomics in kidney disease: From technical approaches to clinical applications” explains the proteomics-based studies on exosomes and their clinical applications in kidney-related diseases (AKI, CKD, renal transplantation, congenital kidney disease, and malignant kidney disorder) (Brahmadhi et al., 2022). Cluster #34 was “human islet and extracellular vesicle.” This article used the potential of human islet-derived EVs in modulating T- and B-cell response activation by EVs in the peripheral blood mononuclear cells (PBMCs) of patients with type 1 diabetes (T1D) (Rutman et al., 2018) (Supplementary Table S4).

### Limitations

Although our study used a comprehensive retrieval strategy to search for data from WoSCC, several limitations should be considered. First, despite conducting the search strategy to the greatest extent by separating “EV-related kidney diseases,” all search samples were extracted by MeSH as search terms. However, fewer studies about EV-related cancer were included in the analysis, which might have caused some inconsistencies in our results. Second, machine algorithms (VOSviewer, CiteSpace, the R Bibliometrix Package, and Excel) were used, and most results might have a few deviations. Lastly, some recent power-published and potentially high-impact studies might not have been included in our research because of the low citation frequency. As a result, more bibliometric data updates would be required to further clarify the scientific trends and hotspots in EV-related kidney disease research.

## Conclusion

To the best of our knowledge, our research is the first to provide a recent assessment of the emerging global trends on EVs in research on kidney-related diseases using a bibliometric approach. Research on EVs in kidney-related diseases is rapidly growing and likely to increase further in the next decade. In conclusion, this study disseminates a comprehensive analysis of EVs in research on kidney-related diseases, providing important information for investigators to formulate new diagnostic, therapeutic, and prognostic ideas or methods in kidney-related diseases.

## Data Availability

The datasets presented in this study can be found in online repositories. The names of the repository/repositories and accession number(s) can be found in the article/Supplementary Material.
