# Supplementary material for: Bibliometric analysis of scientific papers on extracellular vesicles in kidney disease published between 1999 and 2022
Source: Front Cell Dev Biol. 2023 Jan 5;10:1070516. doi: 10.3389/fcell.2022.1070516 (PMC9849820; doi:10.3389/fcell.2022.1070516)
Supplement: Supplementary file 1 [file Table1.docx]

| Kinds | Terms |
| --- | --- |
| Overall  [Kidney related diseases]  **AND {[nanomedicine] OR [extracellular vesicle]}** | #1=[Kidney related diseases]  TS=(“Chronic Kidney Failure” OR “Chronic Renal Insufficiency” OR “chronic kidney disease*” OR “renal failure*” OR “kidney failure” OR “renal impairment” OR “kidney impairment” OR “kidney dysfunction” OR “renal dysfunction” OR “reduced renal function” OR “CKD” OR “progressive kidney” OR “glomerular filtration rate” OR “GFR” OR “eGFR” OR proteinuri* OR “albuminuria” OR “microalbuminuria” OR “End Stage renal disease” OR “ESRD” OR “End-stage Kidney Disease” OR “ESKD” OR “dialysis” OR “Renal Replacement Therapy” OR “kidney transplant” OR “lupus nephritis” OR "acute kidney injuries" OR "acute renal injury" OR "acute renal injuries" OR "acute renal insufficiencies" OR "acute kidney insufficiencies" OR "acute kidney insufficiency" OR "acute renal failure" OR "acute renal failures" OR “AKI” OR “systemic lupus erythematosus” OR “SLE” OR “nephrotic syndrome” OR “NS” OR “glomerulonephritides” OR "iga glomerulonephritis" OR "iga nephropathy" OR "nephritis")  [621,359](https://www.webofscience.com/wos/woscc/summary/af65c30c-6922-4aae-b0a3-4064763a6015-401516b8/relevance/1)  <https://www.webofscience.com/wos/woscc/summary/af65c30c-6922-4aae-b0a3-4064763a6015-401516b8/relevance/1>  #**2=[extracellular vesicle]**  TS=(“extracellular vesicle” OR “vesicle, extracellular” OR “vesicles, extracellular” OR “exovesicles” OR “exovesicle” OR “exosomes” OR “apoptotic bodies” OR “apoptotic body” OR “bodies, apoptotic” OR “body, apoptotic”). [31,299](https://www.webofscience.com/wos/woscc/summary/9134514f-e3ce-49d8-b611-4691d6092876-40152534/relevance/1)  From 1999-2022/6/26 19:00 |
| [Kidney related diseases]  **AND [extracellular vesicle]** | #1 AND #2  [1,122](https://www.webofscience.com/wos/woscc/summary/20cea9ef-9ae5-4910-adfb-eafc1b25747f-416780e8/relevance/1)  <https://www.webofscience.com/wos/woscc/summary/20cea9ef-9ae5-4910-adfb-eafc1b25747f-416780e8/relevance/1> |

**Supplementary Table S1** search strategies and the annual number of publications related to EVs in kidney disease.

**Supplementary Table S1A** search strategies of publications related to EVs in kidney disease.

**Supplementary Table S1B** The annual number of publications related to EVs in kidney disease.

| Year | Count | % of 1062 | Citation |
| --- | --- | --- | --- |
| 1999 | 6 | 0.38% | 2 |
| 2000 | 18 | 0.28% | 30 |
| 2001 | 21 | 0.47% | 36 |
| 2002 | 11 | 0.94% | 90 |
| 2003 | 14 | 0.66% | 119 |
| 2004 | 12 | 0.75% | 191 |
| 2005 | 9 | 0.75% | 237 |
| 2006 | 13 | 0.85% | 258 |
| 2007 | 6 | 0.85% | 330 |
| 2008 | 26 | 1.32% | 426 |
| 2009 | 17 | 1.04% | 457 |
| 2010 | 12 | 1.41% | 542 |
| 2011 | 16 | 1.69% | 690 |
| 2012 | 19 | 2.07% | 825 |
| 2013 | 19 | 3.39% | 1192 |
| 2014 | 28 | 3.48% | 1475 |
| 2015 | 28 | 3.11% | 1861 |
| 2016 | 35 | 6.03% | 2401 |
| 2017 | 32 | 7.16% | 2950 |
| 2018 | 48 | 8.76% | 3908 |
| 2019 | 87 | 11.11% | 5103 |
| 2020 | 95 | 16.76% | 7229 |
| 2021 | 167 | 17.80% | 9748 |
| 2022 | 138 | 8.95% | 4478 |
