# Supplementary material for: Bibliometric analysis of scientific papers on extracellular vesicles in kidney disease published between 1999 and 2022
Source: Front Cell Dev Biol. 2023 Jan 5;10:1070516. doi: 10.3389/fcell.2022.1070516 (PMC9849820; doi:10.3389/fcell.2022.1070516)
Supplement: Supplementary file 2 [file Table2.docx]

The 130 co-cited references at least 20 times for EVs in kidney disease.

| No | Co-cited references | Total link strength | Citations |
| --- | --- | --- | --- |
| 1 | akers jc, 2013, j neuro-oncol, v113, p1, doi 10.1007/s11060-013-1084-8 | 297 | 30 |
| 2 | al-nedawi k, 2008, nat cell biol, v10, p619, doi 10.1038/ncb1725 | 398 | 45 |
| 3 | al-nedawi k, 2009, p natl acad sci usa, v106, p3794, doi 10.1073/pnas.0804543106 | 294 | 34 |
| 4 | alvarez ml, 2012, kidney int, v82, p1024, doi 10.1038/ki.2012.256 | 280 | 35 |
| 5 | alvarez s, 2013, transpl p, v45, p3719, doi 10.1016/j.transproceed.2013.08.079 | 342 | 20 |
| 6 | alvarez-erviti l, 2011, nat biotechnol, v29, p341, doi 10.1038/nbt.1807 | 488 | 52 |
| 7 | arroyo jd, 2011, p natl acad sci usa, v108, p5003, doi 10.1073/pnas.1019055108 | 158 | 24 |
| 8 | balaj l, 2011, nat commun, v2, p0, doi 10.1038/ncomms1180 | 222 | 22 |
| 9 | bartel dp, 2004, cell, v116, p281, doi 10.1016/s0092-8674(04)00045-5 | 132 | 26 |
| 10 | barutta f, 2013, plos one, v8, p0, doi 10.1371/journal.pone.0073798 | 559 | 45 |
| 11 | boilard e, 2010, science, v327, p580, doi 10.1126/science.1181928 | 241 | 21 |
| 12 | borges ft, 2013, j am soc nephrol, v24, p385, doi 10.1681/asn.2012101031 | 353 | 31 |
| 13 | botto m, 1998, nat genet, v19, p56, doi 10.1038/ng0598-56 | 26 | 22 |
| 14 | bruno s, 2009, j am soc nephrol, v20, p1053, doi 10.1681/asn.2008070798 | 698 | 62 |
| 15 | bruno s, 2012, plos one, v7, p0, doi 10.1371/journal.pone.0033115 | 510 | 41 |
| 16 | burger d, 2014, j am soc nephrol, v25, p1401, doi 10.1681/asn.2013070763 | 346 | 24 |
| 17 | buzas ei, 2014, nat rev rheumatol, v10, p356, doi 10.1038/nrrheum.2014.19 | 177 | 22 |
| 18 | camussi g, 2010, kidney int, v78, p838, doi 10.1038/ki.2010.278 | 511 | 49 |
| 19 | cantaluppi v, 2012, kidney int, v82, p412, doi 10.1038/ki.2012.105 | 486 | 37 |
| 20 | casciolarosen la, 1994, j exp med, v179, p1317, doi 10.1084/jem.179.4.1317 | 67 | 36 |
| 21 | cheng l, 2014, kidney int, v86, p433, doi 10.1038/ki.2013.502 | 298 | 33 |
| 22 | cheruvanky a, 2007, am j physiol-renal, v292, pf1657, doi 10.1152/ajprenal.00434.2006 | 341 | 30 |
| 23 | cloutier n, 2013, embo mol med, v5, p235, doi 10.1002/emmm.201201846 | 243 | 23 |
| 24 | cocucci e, 2009, trends cell biol, v19, p43, doi 10.1016/j.tcb.2008.11.003 | 322 | 36 |
| 25 | collino f, 2015, j am soc nephrol, v26, p2349, doi 10.1681/asn.2014070710 | 462 | 36 |
| 26 | colombo m, 2014, annu rev cell dev bi, v30, p255, doi 10.1146/annurev-cellbio-101512-122326 | 953 | 91 |
| 27 | costa-silva b, 2015, nat cell biol, v17, p816, doi 10.1038/ncb3169 | 216 | 25 |
| 28 | dear jw, 2013, proteomics, v13, p1572, doi 10.1002/pmic.201200285 | 325 | 28 |
| 29 | delic d, 2016, plos one, v11, p0, doi 10.1371/journal.pone.0150154 | 206 | 21 |
| 30 | deregibus mc, 2007, blood, v110, p2440, doi 10.1182/blood-2007-03-078709 | 302 | 20 |
| 31 | dominguez jh, 2017, j am soc nephrol, v28, p3533, doi 10.1681/asn.2016121278 | 233 | 20 |
| 32 | dominici m, 2006, cytotherapy, v8, p315, doi 10.1080/14653240600855905 | 147 | 20 |
| 33 | eirin a, 2017, kidney int, v92, p114, doi 10.1016/j.kint.2016.12.023 | 270 | 28 |
| 34 | el andaloussis, 2013, nat rev drug discov, v12, p348, doi 10.1038/nrd3978 | 465 | 47 |
| 35 | erdbrugger u, 2016, j am soc nephrol, v27, p12, doi 10.1681/asn.2015010074 | 415 | 33 |
| 36 | fernandez-llama p, 2010, kidney int, v77, p736, doi 10.1038/ki.2009.550 | 326 | 35 |
| 37 | gatti s, 2011, nephrol dial transpl, v26, p1474, doi 10.1093/ndt/gfr015 | 536 | 44 |
| 38 | gildea jj, 2014, clin biochem, v47, p89, doi 10.1016/j.clinbiochem.2014.06.018 | 401 | 22 |
| 39 | gonzales pa, 2009, j am soc nephrol, v20, p363, doi 10.1681/asn.2008040406 | 541 | 58 |
| 40 | gyorgy b, 2011, cell mol life sci, v68, p2667, doi 10.1007/s00018-011-0689-3 | 419 | 35 |
| 41 | he j, 2012, nephrology, v17, p493, doi 10.1111/j.1440-1797.2012.01589.x | 264 | 20 |
| 42 | heijnen hfg, 1999, blood, v94, p3791, doi 10.1182/blood.v94.11.3791.423a22_3791_3799 | 205 | 20 |
| 43 | hessvik np, 2018, cell mol life sci, v75, p193, doi 10.1007/s00018-017-2595-9 | 207 | 27 |
| 44 | higginbotham jn, 2011, curr biol, v21, p779, doi 10.1016/j.cub.2011.03.043 | 136 | 21 |
| 45 | hogan mc, 2009, j am soc nephrol, v20, p278, doi 10.1681/asn.2008060564 | 476 | 31 |
| 46 | hogan mc, 2014, kidney int, v85, p1225, doi 10.1038/ki.2013.422 | 289 | 21 |
| 47 | hoorn ej, 2005, nephrology, v10, p283, doi 10.1111/j.1440-1797.2005.00387.x | 188 | 21 |
| 48 | hoshino a, 2015, nature, v527, p329, doi 10.1038/nature15756 | 392 | 51 |
| 49 | jeppesen dk, 2019, cell, v177, p428, doi 10.1016/j.cell.2019.02.029 | 279 | 30 |
| 50 | jiang zz, 2016, stem cell res ther, v7, p0, doi 10.1186/s13287-016-0287-2 | 322 | 28 |
| 51 | johnstone rm, 1987, j biol chem, v262, p9412 | 332 | 32 |
| 52 | kahlert c, 2014, j biol chem, v289, p3869, doi 10.1074/jbc.c113.532267 | 276 | 32 |
| 53 | kalani a, 2013, plos one, v8, p0, doi 10.1371/journal.pone.0060177 | 351 | 22 |
| 54 | kalluri r, 2020, science, v367, p640, doi 10.1126/science.aau6977 | 223 | 46 |
| 55 | kamerkar s, 2017, nature, v546, p498, doi 10.1038/nature22341 | 231 | 23 |
| 56 | kapustin an, 2015, circ res, v116, p1312, doi 10.1161/circresaha.116.305012 | 68 | 20 |
| 57 | karpman d, 2017, nat rev nephrol, v13, p545, doi 10.1038/nrneph.2017.98 | 356 | 29 |
| 58 | keller s, 2011, j transl med, v9, p0, doi 10.1186/1479-5876-9-86 | 211 | 24 |
| 59 | kerr jfr, 1972, brit j cancer, v26, p239, doi 10.1038/bjc.1972.33 | 53 | 20 |
| 60 | kowal j, 2014, curr opin cell biol, v29, p116, doi 10.1016/j.ceb.2014.05.004 | 185 | 20 |
| 61 | kowal j, 2016, p natl acad sci usa, v113, pe968, doi 10.1073/pnas.1521230113 | 315 | 34 |
| 62 | lai rc, 2010, stem cell res, v4, p214, doi 10.1016/j.scr.2009.12.003 | 235 | 30 |
| 63 | li zl, 2019, kidney int, v95, p388, doi 10.1016/j.kint.2018.09.013 | 199 | 21 |
| 64 | lin kc, 2016, int j cardiol, v216, p173, doi 10.1016/j.ijcard.2016.04.061 | 208 | 20 |
| 65 | lobb rj, 2015, j extracell vesicles, v4, p0, doi 10.3402/jev.v4.27031 | 151 | 24 |
| 66 | lotvall j, 2014, j extracell vesicles, v3, p0, doi 10.3402/jev.v3.26913 | 503 | 51 |
| 67 | lv ll, 2013, am j physiol-renal, v305, pf1220, doi 10.1152/ajprenal.00148.2013 | 437 | 39 |
| 68 | lv ll, 2013, int j biol sci, v9, p1021, doi 10.7150/ijbs.6100 | 215 | 23 |
| 69 | lv ll, 2020, cell death differ, v27, p210, doi 10.1038/s41418-019-0349-y | 190 | 22 |
| 70 | mathieu m, 2019, nat cell biol, v21, p9, doi 10.1038/s41556-018-0250-9 | 244 | 21 |
| 71 | mathivanan s, 2010, j proteomics, v73, p1907, doi 10.1016/j.jprot.2010.06.006 | 477 | 46 |
| 72 | melo sa, 2015, nature, v523, p177, doi 10.1038/nature14581 | 199 | 34 |
| 73 | merchant ml, 2017, nat rev nephrol, v13, p731, doi 10.1038/nrneph.2017.148 | 213 | 25 |
| 74 | miranda kc, 2010, kidney int, v78, p191, doi 10.1038/ki.2010.106 | 666 | 64 |
| 75 | mitchell ps, 2008, p natl acad sci usa, v105, p10513, doi 10.1073/pnas.0804549105 | 225 | 33 |
| 76 | moon pg, 2011, proteomics, v11, p2459, doi 10.1002/pmic.201000443 | 464 | 34 |
| 77 | morigi m, 2004, j am soc nephrol, v15, p1794, doi 10.1097/01.asn.0000128974.07460.34 | 139 | 20 |
| 78 | mulcahy la, 2014, j extracell vesicles, v3, p0, doi 10.3402/jev.v3.24641 | 422 | 33 |
| 79 | nagaishi k, 2016, sci rep-uk, v6, p0, doi 10.1038/srep34842 | 160 | 22 |
| 80 | nassar wael, 2016, biomater res, v20, p21, doi 10.1186/s40824-016-0068-0 | 290 | 24 |
| 81 | nielsen ct, 2011, arthritis rheum-us, v63, p3067, doi 10.1002/art.30499 | 268 | 22 |
| 82 | nielsen ct, 2012, arthritis rheum-us, v64, p1227, doi 10.1002/art.34381 | 302 | 25 |
| 83 | ohno s, 2013, mol ther, v21, p185, doi 10.1038/mt.2012.180 | 359 | 30 |
| 84 | ostrowski m, 2010, nat cell biol, v12, p19, doi 10.1038/ncb2000 | 275 | 28 |
| 85 | parolini i, 2009, j biol chem, v284, p34211, doi 10.1074/jbc.m109.041152 | 290 | 20 |
| 86 | peinado h, 2012, nat med, v18, p883, doi 10.1038/nm.2753 | 329 | 50 |
| 87 | perez-hernandez j, 2015, plos one, v10, p0, doi 10.1371/journal.pone.0138618 | 291 | 34 |
| 88 | pisitkun t, 2004, p natl acad sci usa, v101, p13368, doi 10.1073/pnas.0403453101 | 1028 | 123 |
| 89 | rabinowits g, 2009, clin lung cancer, v10, p42, doi 10.3816/clc.2009.n.006 | 196 | 32 |
| 90 | raposo g, 1996, j exp med, v183, p1161, doi 10.1084/jem.183.3.1161 | 351 | 34 |
| 91 | raposo g, 2013, j cell biol, v200, p373, doi 10.1083/jcb.201211138 | 643 | 97 |
| 92 | ratajczak j, 2006, leukemia, v20, p1487, doi 10.1038/sj.leu.2404296 | 256 | 22 |
| 93 | ratajczak j, 2006, leukemia, v20, p847, doi 10.1038/sj.leu.2404132 | 244 | 23 |
| 94 | robbins pd, 2014, nat rev immunol, v14, p195, doi 10.1038/nri3622 | 191 | 29 |
| 95 | rood im, 2010, kidney int, v78, p810, doi 10.1038/ki.2010.262 | 349 | 27 |
| 96 | salih m, 2014, am j physiol-renal, v306, pf1251, doi 10.1152/ajprenal.00128.2014 | 302 | 26 |
| 97 | simons m, 2009, curr opin cell biol, v21, p575, doi 10.1016/j.ceb.2009.03.007 | 234 | 30 |
| 98 | simpson rj, 2009, expert rev proteomic, v6, p267, doi 10.1586/epr.09.17 | 211 | 26 |
| 99 | skog j, 2008, nat cell biol, v10, p1470, doi 10.1038/ncb1800 | 487 | 64 |
| 100 | sole c, 2015, nephrol dial transpl, v30, p1488, doi 10.1093/ndt/gfv128 | 457 | 46 |
| 101 | sonoda h, 2009, am j physiol-renal, v297, pf1006, doi 10.1152/ajprenal.00200.2009 | 387 | 21 |
| 102 | street jm, 2011, j physiol-london, v589, p6119, doi 10.1113/jphysiol.2011.220277 | 391 | 25 |
| 103 | taylor dd, 2008, gynecol oncol, v110, p13, doi 10.1016/j.ygyno.2008.04.033 | 290 | 32 |
| 104 | thery c, 2001, j immunol, v166, p7309, doi 10.4049/jimmunol.166.12.7309 | 150 | 20 |
| 105 | thery c, 2002, nat rev immunol, v2, p569, doi 10.1038/nri855 | 475 | 71 |
| 106 | thery c, 2009, nat rev immunol, v9, p581, doi 10.1038/nri2567 | 695 | 89 |
| 107 | thery c, 2018, j extracell vesicles, v7, p0, doi 10.1080/20013078.2018.1535750 | 472 | 60 |
| 108 | thery clotilde, 2006, curr protoc cell biol, vchapter 3, p0, doi 10.1002/0471143030.cb0322s30 | 567 | 69 |
| 109 | tkach m, 2016, cell, v164, p1226, doi 10.1016/j.cell.2016.01.043 | 390 | 51 |
| 110 | tomasoni s, 2013, stem cells dev, v22, p0, doi 10.1089/scd.2012.0266 | 419 | 25 |
| 111 | trajkovic k, 2008, science, v319, p1244, doi 10.1126/science.1153124 | 328 | 32 |
| 112 | valadi h, 2007, nat cell biol, v9, p654, doi 10.1038/ncb1596 | 1273 | 158 |
| 113 | van balkombwm, 2011, kidney int, v80, p1138, doi 10.1038/ki.2011.292 | 298 | 38 |
| 114 | van derpole, 2012, pharmacol rev, v64, p676, doi 10.1124/pr.112.005983 | 320 | 31 |
| 115 | van nielg, 2018, nat rev mol cell bio, v19, p213, doi 10.1038/nrm.2017.125 | 445 | 55 |
| 116 | vinas jl, 2016, kidney int, v90, p1238, doi 10.1016/j.kint.2016.07.015 | 210 | 25 |
| 117 | vlassov av, 2012, bba-gen subjects, v1820, p940, doi 10.1016/j.bbagen.2012.03.017 | 201 | 35 |
| 118 | wang b, 2016, mol ther, v24, p1290, doi 10.1038/mt.2016.90 | 352 | 33 |
| 119 | williams c, 2014, cell res, v24, p766, doi 10.1038/cr.2014.44 | 327 | 52 |
| 120 | witwer kw, 2013, j extracell vesicles, v2, p0, doi 10.3402/jev.v2i0.20360 | 435 | 39 |
| 121 | yanez-mo m, 2015, j extracell vesicles, v4, p0, doi 10.3402/jev.v4.27066 | 640 | 69 |
| 122 | zhang hy, 2017, nat commun, v8, p0, doi 10.1038/ncomms15016 | 120 | 20 |
| 123 | zhang j, 2015, genom proteom bioinf, v13, p17, doi 10.1016/j.gpb.2015.02.001 | 130 | 23 |
| 124 | zhang w, 2016, am j physiol-renal, v311, pf844, doi 10.1152/ajprenal.00429.2016 | 199 | 23 |
| 125 | zhou h, 2006, kidney int, v69, p1471, doi 10.1038/sj.ki.5000273 | 214 | 30 |
| 126 | zhou h, 2006, kidney int, v70, p1847, doi 10.1038/sj.ki.5001874 | 442 | 32 |
| 127 | zhou h, 2008, kidney int, v74, p613, doi 10.1038/ki.2008.206 | 521 | 39 |
| 128 | zhou y, 2013, stem cell res ther, v4, p0, doi 10.1186/scrt194 | 385 | 35 |
| 129 | zou xy, 2014, stem cell res ther, v5, p0, doi 10.1186/scrt428 | 358 | 25 |
| 130 | zubiri i, 2014, j proteomics, v96, p92, doi 10.1016/j.jprot.2013.10.037 | 369 | 28 |
