# Supplementary material for: Bibliometric analysis of scientific papers on extracellular vesicles in kidney disease published between 1999 and 2022
Source: Front Cell Dev Biol. 2023 Jan 5;10:1070516. doi: 10.3389/fcell.2022.1070516 (PMC9849820; doi:10.3389/fcell.2022.1070516)
Supplement: Supplementary file 3 [file Table3.docx]

| **From** | **To** | **Words** | **Weighted Inclusion Index** | **Inclusion Index** | **Occurrences** | **Stability Index** |
| --- | --- | --- | --- | --- | --- | --- |
| apoptosis--1999-2007 | apoptosis--2008-2013 | apoptosis | 0.82 | 0.50 | 25 | 0.13 |
| apoptosis--1999-2007 | autoimmunity--2008-2013 | autoimmunity;phagocytosis | 0.75 | 0.33 | 4 | 0.11 |
| autoantibodies--1999-2007 | autoantibodies--2008-2013 | autoantibodies | 1.00 | 1.00 | 3 | 1.00 |
| biomarker--1999-2007 | chronic kidney disease--2008-2013 | urine | 0.50 | 0.50 | 2 | 0.11 |
| biomarker--1999-2007 | exosome--2008-2013 | biomarker | 0.50 | 0.50 | 2 | 0.13 |
| mass spectrometry--1999-2007 | chronic kidney disease--2008-2013 | mass spectrometry | 1.00 | 1.00 | 2 | 0.13 |
| systemic lupus erythematosus--1999-2007 | systemic lupus erythematosus--2008-2013 | systemic lupus erythematosus | 1.00 | 1.00 | 4 | 0.50 |
| vascular calcification--1999-2007 | cardiovascular diseases--2008-2013 | vascular calcification | 1.00 | 1.00 | 2 | 0.33 |
| apoptosis--2008-2013 | apoptosis--2014-2018 | apoptosis | 0.59 | 0.50 | 14 | 0.17 |
| apoptosis--2008-2013 | exosomes--2014-2018 | acute kidney injury | 0.18 | 0.50 | 3 | 0.02 |
| autoimmunity--2008-2013 | systemic lupus erythematosus--2014-2018 | autoimmunity | 0.50 | 0.33 | 4 | 0.07 |
| cardiovascular diseases--2008-2013 | exosomes--2014-2018 | extracellular vesicles | 0.33 | 0.33 | 2 | 0.02 |
| cardiovascular diseases--2008-2013 | vascular calcification--2014-2018 | vascular calcification | 0.40 | 1.00 | 2 | 0.33 |
| chronic kidney disease--2008-2013 | exosomes--2014-2018 | chronic kidney disease;urine;diabetic nephropathy;biomarkers;epigenetics;urinary exosomes | 0.83 | 0.13 | 6 | 0.02 |
| exosome--2008-2013 | exosomes--2014-2018 | exosome;biomarker;proteomics;microrna | 0.74 | 0.14 | 13 | 0.02 |
| exosomes--2008-2013 | angiogenesis--2014-2018 | breast cancer | 0.12 | 0.17 | 2 | 0.07 |
| exosomes--2008-2013 | exosomes--2014-2018 | exosomes;targeted therapy | 0.46 | 0.11 | 15 | 0.02 |
| exosomes--2008-2013 | liquid biopsy--2014-2018 | cancer;egfr;oncogenes | 0.27 | 0.11 | 4 | 0.04 |
| inflammation--2008-2013 | aldosterone--2014-2018 | placenta | 0.17 | 0.20 | 2 | 0.11 |
| inflammation--2008-2013 | exosomes--2014-2018 | microvesicles;hypertension | 0.42 | 0.20 | 3 | 0.02 |
| inflammation--2008-2013 | systemic lupus erythematosus--2014-2018 | inflammation | 0.25 | 0.20 | 3 | 0.06 |
| kidney--2008-2013 | exosomes--2014-2018 | kidney | 1.00 | 1.00 | 2 | 0.02 |
| microparticles--2008-2013 | systemic lupus erythematosus--2014-2018 | microparticles | 1.00 | 1.00 | 2 | 0.08 |
| systemic lupus erythematosus--2008-2013 | systemic lupus erythematosus--2014-2018 | systemic lupus erythematosus | 1.00 | 1.00 | 4 | 0.08 |
| aldosterone--2014-2018 | preeclampsia--2019-2022 | proteinuria;preeclampsia | 0.42 | 0.20 | 3 | 0.08 |
| angiogenesis--2014-2018 | cancer--2019-2022 | metastasis | 0.18 | 0.17 | 3 | 0.07 |
| angiogenesis--2014-2018 | egfr--2019-2022 | angiogenesis;glioma | 0.29 | 0.17 | 3 | 0.04 |
| angiogenesis--2014-2018 | glioblastoma--2019-2022 | glioblastoma | 0.30 | 0.25 | 3 | 0.11 |
| apoptosis--2014-2018 | cancer--2019-2022 | tumor microenvironment | 0.11 | 0.20 | 2 | 0.08 |
| apoptosis--2014-2018 | exosomes--2019-2022 | apoptosis;cell death;macrophage | 0.78 | 0.20 | 10 | 0.01 |
| apoptosis--2014-2018 | systemic lupus erythematosus--2019-2022 | pathogenesis | 0.11 | 0.20 | 2 | 0.05 |
| apoptotic bodies--2014-2018 | egfr--2019-2022 | reactive oxygen species | 0.50 | 0.50 | 2 | 0.05 |
| apoptotic bodies--2014-2018 | exosomes--2019-2022 | apoptotic bodies | 0.50 | 0.50 | 2 | 0.01 |
| circulating tumor dna (ctdna)--2014-2018 | extracellular vesicles (ev)--2019-2022 | non-small cell lung cancer (nsclc) | 0.38 | 0.33 | 3 | 0.17 |
| drug delivery--2014-2018 | systemic lupus erythematosus--2019-2022 | drug delivery | 0.22 | 0.25 | 3 | 0.05 |
| epidermal growth factor receptor--2014-2018 | liquid biopsy--2019-2022 | epidermal growth factor receptor;cetuximab;colorectal cancer | 0.50 | 0.14 | 5 | 0.05 |
| epidermal growth factor receptor--2014-2018 | systemic lupus erythematosus--2019-2022 | epithelial-mesenchymal transition | 0.10 | 0.14 | 2 | 0.04 |
| exosomes--2014-2018 | cancer--2019-2022 | prostate cancer | 0.06 | 0.11 | 2 | 0.02 |
| exosomes--2014-2018 | egfr--2019-2022 | drug resistance | 0.02 | 0.06 | 2 | 0.02 |
| exosomes--2014-2018 | exosomes--2019-2022 | exosomes;extracellular vesicles;exosome;microrna;biomarker;microvesicles;urine;biomarkers;extracellular vesicle;mesenchymal stem cells;chronic kidney disease;kidney;diabetic nephropathy;proteomics;renal fibrosis;mirna;micrornas;podocyte;stem cells;hypertension;acute kidney injury;autophagy;therapy;nephrotoxicity;ckd;diagnosis;glomerular disease;iga nephropathy | 0.85 | 0.02 | 84 | 0.01 |
| exosomes--2014-2018 | fibrosis--2019-2022 | fibrosis | 0.11 | 0.25 | 2 | 0.02 |
| exosomes--2014-2018 | systemic lupus erythematosus--2019-2022 | epigenetics | 0.02 | 0.06 | 2 | 0.02 |
| exosomes--2014-2018 | urinary exosomes--2019-2022 | albuminuria;urinary exosomes;aquaporin-2;diabetic kidney disease;ultracentrifugation | 0.20 | 0.06 | 5 | 0.02 |
| exosomes--2014-2018 | vascular calcification--2019-2022 | ageing | 0.07 | 0.17 | 2 | 0.02 |
| flow cytometry--2014-2018 | exosomes--2019-2022 | sepsis | 0.29 | 0.33 | 2 | 0.01 |
| kidney transplantation--2014-2018 | exosomes--2019-2022 | kidney transplantation | 1.00 | 1.00 | 2 | 0.01 |
| liquid biopsy--2014-2018 | cancer--2019-2022 | cancer | 0.26 | 0.11 | 11 | 0.04 |
| liquid biopsy--2014-2018 | egfr--2019-2022 | egfr;lung cancer;nsclc | 0.33 | 0.06 | 17 | 0.03 |
| liquid biopsy--2014-2018 | exosomes--2019-2022 | mirnas | 0.05 | 0.06 | 5 | 0.01 |
| liquid biopsy--2014-2018 | liquid biopsy--2019-2022 | liquid biopsy;non-small cell lung cancer;circulating tumor cells;ctdna;circulating tumor dna | 0.43 | 0.07 | 20 | 0.03 |
| systemic lupus erythematosus--2014-2018 | egfr--2019-2022 | macrophages | 0.06 | 0.08 | 3 | 0.03 |
| systemic lupus erythematosus--2014-2018 | exosomes--2019-2022 | inflammation;cell therapy;mesenchymal stem cell;plasma | 0.35 | 0.08 | 12 | 0.01 |
| systemic lupus erythematosus--2014-2018 | systemic lupus erythematosus--2019-2022 | systemic lupus erythematosus;autoimmunity;microparticles;rheumatoid arthritis | 0.47 | 0.08 | 13 | 0.04 |
| vascular calcification--2014-2018 | vascular calcification--2019-2022 | vascular calcification | 1.00 | 1.00 | 5 | 0.17 |

**Supplementary Table 3** Major keywords evolution of EVs in kidney disease research.
