# Supplementary material for: Bibliometric analysis of scientific papers on extracellular vesicles in kidney disease published between 1999 and 2022
Source: Front Cell Dev Biol. 2023 Jan 5;10:1070516. doi: 10.3389/fcell.2022.1070516 (PMC9849820; doi:10.3389/fcell.2022.1070516)
Supplement: Supplementary file 4 [file Table4.docx]

**Co-cited keywords related results**

The network is divided into **13** co-citation clusters. The largest **13** clusters are summarized as follows.

**Table 1. Summary of the largest 13 clusters.**

| **ClusterID** | **Size** | **Silhouette** | **Label (LSI)** | **Label (LLR)** | **Label (MI)** | **Average Year** |
| --- | --- | --- | --- | --- | --- | --- |
| 0 | 168 | 0.816 | extracellular vesicle | urinary exosome (328.35, 1.0E-4) | stem cell-derived (1.48) | 2014 |
| 1 | 152 | 0.872 | extracellular vesicle | acute kidney injury (461.54, 1.0E-4) | stem cell-derived (2.17) | 2020 |
| 2 | 125 | 0.87 | liquid biopsy | liquid biopsy (513.13, 1.0E-4) | kidney regeneration (3.6) | 2017 |
| 4 | 70 | 0.936 | extracellular vesicle | membrane vesicle (153.34, 1.0E-4) | breast cancer cell (0.12) | 2012 |
| 5 | 63 | 0.97 | breast cancer | extracellular nucleic acid (83.63, 1.0E-4) | extracellular vesicle (0.08) | 2009 |
| 6 | 44 | 0.933 | extracellular vesicle | mesenchymal stem (145.75, 1.0E-4) | stem cell-derived (0.11) | 2016 |
| 7 | 31 | 0.937 | systemic lupus erythematosus | systemic lupus erythematosus (237.96, 1.0E-4) | exosome-based drug delivery system (0.11) | 2020 |
| 10 | 23 | 0.934 | extracellular vesicle | recombinant phosphatidylserine-binding nanobodies (133.24, 1.0E-4) | stem cell-derived (0.21) | 2017 |
| 13 | 10 | 0.988 | mesenchymal stem | therapeutic vehicle (51.77, 1.0E-4) | extracellular vesicle (0.08) | 2022 |
| 18 | 7 | 0.994 | peritoneal dialysis | novel biomarker source (42.83, 1.0E-4) | extracellular vesicle (0.08) | 2022 |
| 23 | 5 | 0.994 | vascular calcification | vascular calcification (58.82, 1.0E-4) | extracellular vesicle (0.08) | 2021 |
| 30 | 4 | 1 | isolation and characterization of circulating micro(nano)vesicles in the plasma of colorectal cancer patients and their interactions with tumor cells | plasma (16.47, 1.0E-4) | extracellular vesicle (0.09) | 2015 |
| 34 | 3 | 0.996 | extracellular vesicle | human islet (67.76, 1.0E-4) | extracellular vesicle (0.08) | 2017 |

The largest cluster (#0) has 168 members and a silhouette value of 0.816. It is labeled as  *urinary exosome* by LLR, *extracellular vesicle* by LSI, and *stem cell-derived (1.48)* by MI. The most relevant citer to the cluster is ERDBRUEGGER, U (2016.0) [Extracellular vesicles in renal diseases: more than novel biomarkers?](http://dx.doi.org/10.1681/ASN.2015010074). JOURNAL OF THE AMERICAN SOCIETY OF NEPHROLOGY, V27, P15 DOI 10.1681/ASN.2015010074.

The second largest cluster (#1) has 152 members and a silhouette value of 0.872. It is labeled as  *acute kidney injury* by LLR, *extracellular vesicle* by LSI, and *stem cell-derived (2.17)* by MI. The most relevant citer to the cluster is JIN, C (2021.0) [Exosomes: emerging therapy delivery tools and biomarkers for kidney diseases](http://dx.doi.org/10.1155/2021/7844455). STEM CELLS INTERNATIONAL, V2021, P18 DOI 10.1155/2021/7844455.

The third largest cluster (#2) has 125 members and a silhouette value of 0.87. It is labeled as ***liquid biopsy*** by both LLR and LSI, and as *kidney regeneration (3.6)* by MI. The most relevant citer to the cluster is CHOI, D (2017.0) [Extracellular vesicle communication pathways as regulatory targets of oncogenic transformation](http://dx.doi.org/10.1016/j.semcdb.2017.01.003). SEMINARS IN CELL & DEVELOPMENTAL BIOLOGY, V67, P12 DOI 10.1016/j.semcdb.2017.01.003.

The 4th largest cluster (#4) has 70 members and a silhouette value of 0.936. It is labeled as  *membrane vesicle* by LLR, *extracellular vesicle* by LSI, and *breast cancer cell (0.12)* by MI. The most relevant citer to the cluster is GYOERGY, B (2011.0) [Membrane vesicles, current state-of-the-art: emerging role of extracellular vesicles](http://dx.doi.org/10.1007/s00018-011-0689-3). CELLULAR AND MOLECULAR LIFE SCIENCES, V68, P22 DOI 10.1007/s00018-011-0689-3.

The 5th largest cluster (#5) has 63 members and a silhouette value of 0.97. It is labeled as  *extracellular nucleic acid* by LLR, *breast cancer* by LSI, and *extracellular vesicle (0.08)* by MI. The most relevant citer to the cluster is FRIEL, A (2010.0) [Relevance of circulating tumor cells, extracellular nucleic acids, and exosomes in breast cancer](http://dx.doi.org/10.1007/s10549-010-0980-2). BREAST CANCER RESEARCH AND TREATMENT, V123, P13 DOI 10.1007/s10549-010-0980-2.

The 6th largest cluster (#6) has 44 members and a silhouette value of 0.933. It is labeled as  *mesenchymal stem* by LLR, *extracellular vesicle* by LSI, and *stem cell-derived (0.11)* by MI. The most relevant citer to the cluster is NARGESI, A (2017.0) [Mesenchymal stem cell-derived extracellular vesicles for renal repair](http://dx.doi.org/10.2174/1566523217666170412110724). CURRENT GENE THERAPY, V17, P14 DOI 10.2174/1566523217666170412110724.

The 7th largest cluster (#7) has 31 members and a silhouette value of 0.937. It is labeled as ***systemic lupus erythematosus*** by both LLR and LSI, and as *exosome-based drug delivery system (0.11)* by MI. The most relevant citer to the cluster is ORTEGA, A (2021.0) [Exosomes as drug delivery systems: endogenous nanovehicles for treatment of systemic lupus erythematosus](http://dx.doi.org/10.3390/pharmaceutics13010003). PHARMACEUTICS, V13, P28 DOI 10.3390/pharmaceutics13010003.

The 8th largest cluster (#10) has 23 members and a silhouette value of 0.934. It is labeled as  *recombinant phosphatidylserine-binding nanobodies* by LLR, *extracellular vesicle* by LSI, and *stem cell-derived (0.21)* by MI. The most relevant citer to the cluster is KOOIJMANS, S (2016.0) [Pegylated and targeted extracellular vesicles display enhanced cell specificity and circulation time](http://dx.doi.org/10.1016/j.jconrel.2016.01.009). JOURNAL OF CONTROLLED RELEASE DOI 10.1016/j.jconrel.2016.01.009.

The 9th largest cluster (#13) has 10 members and a silhouette value of 0.988. It is labeled as  *therapeutic vehicle* by LLR, *mesenchymal stem* by LSI, and *extracellular vesicle (0.08)* by MI. The most relevant citer to the cluster is MARTINEZ-ARROYO, O (2022.0) [Mesenchymal stem cell-derived extracellular vesicles as non-coding rna therapeutic vehicles in autoimmune diseases](http://dx.doi.org/10.3390/pharmaceutics14040733). PHARMACEUTICS, V14, P30 DOI 10.3390/pharmaceutics14040733.

The 10th largest cluster (#18) has 7 members and a silhouette value of 0.994. It is labeled as  *novel biomarker source* by LLR, *peritoneal dialysis* by LSI, and *extracellular vesicle (0.08)* by MI. The most relevant citer to the cluster is BRAHMADHI, A (2022.0) [Exosomal proteomics in kidney disease: from technical approaches to clinical applications](http://dx.doi.org/10.38212/2224-6614.3409). JOURNAL OF FOOD AND DRUG ANALYSIS, V30, P22 DOI 10.38212/2224-6614.3409.

The 11th largest cluster (#23) has 5 members and a silhouette value of 0.994. It is labeled as ***vascular calcification*** by both LLR and LSI, and as *extracellular vesicle (0.08)* by MI. The most relevant citer to the cluster is QIN, Z (2021.0) [A narrative review of exosomes in vascular calcification](http://dx.doi.org/10.21037/atm-20-7355). ANNALS OF TRANSLATIONAL MEDICINE DOI 10.21037/atm-20-7355.

The 12th largest cluster (#30) has 4 members and a silhouette value of 1. It is labeled as  *plasma* by LLR, *vesicles in the plasma of colorectal cancer patients and their interactions with tumor cells* by LSI, and *extracellular vesicle (0.09)* by MI. The most relevant citer to the cluster is STEC, M (2015.0) [Isolation and characterization of circulating micro(nano)vesicles in the plasma of colorectal cancer patients and their interactions with tumor cells](http://dx.doi.org/10.3892/or.2015.4228). ONCOLOGY REPORTS DOI 10.3892/or.2015.4228.

The 13th largest cluster (#34) has 3 members and a silhouette value of 0.996. It is labeled as  *human islet* by LLR, *extracellular vesicle* by LSI, and *extracellular vesicle (0.08)* by MI. The most relevant citer to the cluster is RUTMAN, A (2018.0) [Immune response to extracellular vesicles from human islets of langerhans in patients with type 1 diabetes](http://dx.doi.org/10.1210/en.2018-00649). ENDOCRINOLOGY, V159, P14 DOI 10.1210/en.2018-00649.

## CITATION COUNTS

The top ranked item by citation counts is Thery C (2018) in Cluster #2, with citation counts of **60**. The second one is Van NIELG (2018) in Cluster #2, with citation counts of **53**. The third is Colombo M (2014) in Cluster #2, with citation counts of **49**. The 4th is Yanez-mo M (2015) in Cluster #10, with citation counts of **48**. The 5th is Kalluri R (2020) in Cluster #1, with citation counts of **46**. The 6th is Raposo G (2013) in Cluster #0, with citation counts of **42**. The 7th is Tkach M (2016) in Cluster #2, with citation counts of **42**. The 8th is Hoshino A (2015) in Cluster #2, with citation counts of **39**. The 9th is Williams C (2014) in Cluster #2, with citation counts of **35**. The 10th is Kowal J (2016) in Cluster #2, with citation counts of **31**.

| **Citation Counts** | **References** | **DOI** | **Cluster ID** |
| --- | --- | --- | --- |
| 60 | Thery C, 2018, J EXTRACELL VESICLES, 7, 0 | 10.1080/20013078.2018.1535750 | 2 |
| 53 | Van NIELG, 2018, NAT REV MOL CELL BIO, 19, 213 | 10.1038/nrm.2017.125 | 2 |
| 49 | Colombo M, 2014, ANNU REV CELL DEV BI, 30, 255 | 10.1146/annurev-cellbio-101512-122326 | 2 |
| 48 | Yanez-mo M, 2015, J EXTRACELL VESICLES, 4, 0 | 10.3402/jev.v4.27066 | 10 |
| 46 | Kalluri R, 2020, SCIENCE, 367, 640 | 10.1126/science.aau6977 | 1 |
| 42 | Raposo G, 2013, J CELL BIOL, 200, 373 | 10.1083/jcb.201211138 | 0 |
| 42 | Tkach M, 2016, CELL, 164, 1226 | 10.1016/j.cell.2016.01.043 | 2 |
| 39 | Hoshino A, 2015, NATURE, 527, 329 | 10.1038/nature15756 | 2 |
| 35 | Williams C, 2014, CELL RES, 24, 766 | 10.1038/cr.2014.44 | 2 |
| 31 | Kowal J, 2016, P NATL ACAD SCI USA, 113, 0 | 10.1073/pnas.1521230113 | 2 |

## BURSTS

The top ranked item by bursts is Colombo M (2014) in Cluster #2, with bursts of **18.38**. The second one is Raposo G (2013) in Cluster #0, with bursts of **16.36**. The third is Thery C (2018) in Cluster #2, with bursts of **13.62**. The 4th is Miranda KC (2010) in Cluster #0, with bursts of **13.26**. The 5th is Williams C (2014) in Cluster #2, with bursts of **13.08**. The 6th is Thery C (2009) in Cluster #4, with bursts of **12.49**. The 7th is Hoshino A (2015) in Cluster #2, with bursts of **11.86**. The 8th is Van NIELG (2018) in Cluster #2, with bursts of **11.52**. The 9th is Peinado H (2012) in Cluster #2, with bursts of **11.26**. The 10th is Alvarez ML (2012) in Cluster #0, with bursts of **11.15**.

| **Bursts** | **References** | **DOI** | **Cluster ID** |
| --- | --- | --- | --- |
| 18.38 | Colombo M, 2014, ANNU REV CELL DEV BI, 30, 255 | 10.1146/annurev-cellbio-101512-122326 | 2 |
| 16.36 | Raposo G, 2013, J CELL BIOL, 200, 373 | 10.1083/jcb.201211138 | 0 |
| 13.62 | Thery C, 2018, J EXTRACELL VESICLES, 7, 0 | 10.1080/20013078.2018.1535750 | 2 |
| 13.26 | Miranda KC, 2010, KIDNEY INT, 78, 191 | 10.1038/ki.2010.106 | 0 |
| 13.08 | Williams C, 2014, CELL RES, 24, 766 | 10.1038/cr.2014.44 | 2 |
| 12.49 | Thery C, 2009, NAT REV IMMUNOL, 9, 581 | 10.1038/nri2567 | 4 |
| 11.86 | Hoshino A, 2015, NATURE, 527, 329 | 10.1038/nature15756 | 2 |
| 11.52 | Van NIELG, 2018, NAT REV MOL CELL BIO, 19, 213 | 10.1038/nrm.2017.125 | 2 |
| 11.26 | Peinado H, 2012, NAT MED, 18, 883 | 10.1038/nm.2753 | 2 |
| 11.15 | Alvarez ML, 2012, KIDNEY INT, 82, 1024 | 10.1038/ki.2012.256 | 0 |

## DEGREE

The top ranked item by degree is Ranghino A (2017) in Cluster #1, with degree of **49**. The second one is Al-nedawi K (2008) in Cluster #4, with degree of **43**. The third is Burger D (2014) in Cluster #0, with degree of **40**. The 4th is Zubiri I (2014) in Cluster #0, with degree of **38**. The 5th is Collino F (2015) in Cluster #6, with degree of **37**. The 6th is Wang B (2016) in Cluster #1, with degree of **35**. The 7th is Lin KC (2016) in Cluster #1, with degree of **35**. The 8th is Chen L (2020) in Cluster #1, with degree of **35**. The 9th is Bruno S (2012) in Cluster #6, with degree of **34**. The 10th is Akers JC (2013) in Cluster #0, with degree of **34**.

| **Degree** | **References** | **DOI** | **Cluster ID** |
| --- | --- | --- | --- |
| 49 | Ranghino A, 2017, STEM CELL RES THER, 8, 0 | 10.1186/s13287-017-0478-5 | 1 |
| 43 | Al-nedawi K, 2008, NAT CELL BIOL, 10, 619 | 10.1038/ncb1725 | 4 |
| 40 | Burger D, 2014, J AM SOC NEPHROL, 25, 1401 | 10.1681/ASN.2013070763 | 0 |
| 38 | Zubiri I, 2014, J PROTEOMICS, 96, 92 | 10.1016/j.jprot.2013.10.037 | 0 |
| 37 | Collino F, 2015, J AM SOC NEPHROL, 26, 2349 | 10.1681/ASN.2014070710 | 6 |
| 35 | Wang B, 2016, MOL THER, 24, 1290 | 10.1038/mt.2016.90 | 1 |
| 35 | Lin KC, 2016, INT J CARDIOL, 216, 173 | 10.1016/j.ijcard.2016.04.061 | 1 |
| 35 | Chen L, 2020, THERANOSTICS, 10, 9425 | 10.7150/thno.43315 | 1 |
| 34 | Bruno S, 2012, PLOS ONE, 7, 0 | 10.1371/journal.pone.0033115 | 6 |
| 34 | Akers JC, 2013, J NEURO-ONCOL, 113, 1 | 10.1007/s11060-013-1084-8 | 0 |

## CENTRALITY

The top ranked item by centrality is Burger D (2014) in Cluster #0, with centrality of **0.09**. The second one is Thery C (2009) in Cluster #4, with centrality of **0.07**. The third is Al-nedawi K (2008) in Cluster #4, with centrality of **0.05**. The 4th is Barutta F (2013) in Cluster #0, with centrality of **0.05**. The 5th is Karpman D (2017) in Cluster #1, with centrality of **0.04**. The 6th is Boilard E (2010) in Cluster #0, with centrality of **0.04**. The 7th is Sole C (2015) in Cluster #0, with centrality of **0.04**. The 8th is Delic D (2016) in Cluster #1, with centrality of **0.04**. The 9th is Mitchell PS (2008) in Cluster #5, with centrality of **0.04**. The 10th is Mulcahy LA (2014) in Cluster #10, with centrality of **0.04**.

| **Centrality** | **References** | **DOI** | **Cluster ID** |
| --- | --- | --- | --- |
| 0.09 | Burger D, 2014, J AM SOC NEPHROL, 25, 1401 | 10.1681/ASN.2013070763 | 0 |
| 0.07 | Thery C, 2009, NAT REV IMMUNOL, 9, 581 | 10.1038/nri2567 | 4 |
| 0.05 | Al-nedawi K, 2008, NAT CELL BIOL, 10, 619 | 10.1038/ncb1725 | 4 |
| 0.05 | Barutta F, 2013, PLOS ONE, 8, 0 | 10.1371/journal.pone.0073798 | 0 |
| 0.04 | Karpman D, 2017, NAT REV NEPHROL, 13, 545 | 10.1038/nrneph.2017.98 | 1 |
| 0.04 | Boilard E, 2010, SCIENCE, 327, 580 | 10.1126/science.1181928 | 0 |
| 0.04 | Sole C, 2015, NEPHROL DIAL TRANSPL, 30, 1488 | 10.1093/ndt/gfv128 | 0 |
| 0.04 | Delic D, 2016, PLOS ONE, 11, 0 | 10.1371/journal.pone.0150154 | 1 |
| 0.04 | Mitchell PS, 2008, P NATL ACAD SCI USA, 105, 10513 | 10.1073/pnas.0804549105 | 5 |
| 0.04 | Mulcahy LA, 2014, J EXTRACELL VESICLES, 3, 0 | 10.3402/jev.v3.24641 | 10 |

## SIGMA

The top ranked item by sigma is Thery C (2009) in Cluster #4, with sigma of **2.32**. The second one is Barutta F (2013) in Cluster #0, with sigma of **1.54**. The third is Williams C (2014) in Cluster #2, with sigma of **1.51**. The 4th is Alvarez ML (2012) in Cluster #0, with sigma of **1.46**. The 5th is Burger D (2014) in Cluster #0, with sigma of **1.34**. The 6th is Colombo M (2014) in Cluster #2, with sigma of **1.34**. The 7th is Yanez-mo M (2015) in Cluster #10, with sigma of **1.33**. The 8th is Sole C (2015) in Cluster #0, with sigma of **1.32**. The 9th is Mulcahy LA (2014) in Cluster #10, with sigma of **1.29**. The 10th is Gonzales PA (2009) in Cluster #0, with sigma of **1.29**.

| **Sigma** | **References** | **DOI** | **Cluster ID** |
| --- | --- | --- | --- |
| 2.32 | Thery C, 2009, NAT REV IMMUNOL, 9, 581 | 10.1038/nri2567 | 4 |
| 1.54 | Barutta F, 2013, PLOS ONE, 8, 0 | 10.1371/journal.pone.0073798 | 0 |
| 1.51 | Williams C, 2014, CELL RES, 24, 766 | 10.1038/cr.2014.44 | 2 |
| 1.46 | Alvarez ML, 2012, KIDNEY INT, 82, 1024 | 10.1038/ki.2012.256 | 0 |
| 1.34 | Burger D, 2014, J AM SOC NEPHROL, 25, 1401 | 10.1681/ASN.2013070763 | 0 |
| 1.34 | Colombo M, 2014, ANNU REV CELL DEV BI, 30, 255 | 10.1146/annurev-cellbio-101512-122326 | 2 |
| 1.33 | Yanez-mo M, 2015, J EXTRACELL VESICLES, 4, 0 | 10.3402/jev.v4.27066 | 10 |
| 1.32 | Sole C, 2015, NEPHROL DIAL TRANSPL, 30, 1488 | 10.1093/ndt/gfv128 | 0 |
| 1.29 | Mulcahy LA, 2014, J EXTRACELL VESICLES, 3, 0 | 10.3402/jev.v3.24641 | 10 |
| 1.29 | Gonzales PA, 2009, J AM SOC NEPHROL, 20, 363 | 10.1681/ASN.2008040406 | 0 |

**Co-cited references related results by Citespace**

**REFERENCE of EVs**

Time of creation: Thu Aug 11 03:48:15 CST 2022

## MAJOR CLUSTERS

The network is divided into **10** co-citation clusters. The largest **10** clusters are summarized as follows.

**Table 1. Summary of the largest 10 clusters.**

| **ClusterID** | **Size** | **Silhouette** | **Label (LSI)** | **Label (LLR)** | **Label (MI)** | **Average Year** |
| --- | --- | --- | --- | --- | --- | --- |
| 0 | 130 | 0.761 | extracellular vesicle | apoptotic bodies (534.33, 1.0E-4) | fate (0.25) | 2005 |
| 1 | 99 | 0.729 | liquid biopsy | liquid biopsy (3315.27, 1.0E-4) | fate (1.32) | 2016 |
| 2 | 91 | 0.715 | extracellular vesicle | cell-derived extracellular vesicle (2539.4, 1.0E-4) | fate (3.09) | 2014 |
| 3 | 78 | 0.716 | extracellular vesicle | ultracentrifugation supernatant (442.87, 1.0E-4) | fate (2.16) | 2011 |
| 4 | 62 | 0.811 | vascular calcification | vascular calcification (2366.42, 1.0E-4) | fate (0.27) | 2009 |
| 5 | 56 | 0.797 | systemic lupus erythematosus | cell-derived extracellular vesicle (656.13, 1.0E-4) | fate (1.52) | 2008 |
| 6 | 42 | 0.779 | urinary extracellular vesicle | urinary extracellular vesicle (339.8, 1.0E-4) | mir-451 loaded exosome (0.22) | 2016 |
| 7 | 23 | 0.959 | crystal structure | crystal structure (67.57, 1.0E-4) | extracellular vesicle (0.04) | 2002 |
| 8 | 18 | 0.95 | ion fluxe | cd95 activation (118.91, 1.0E-4) | extracellular vesicle (0.04) | 2008 |
| 10 | 4 | 0.997 | oligonucleotides suppress pkb/akt and act as superinductors of apoptosis in human keratinocytes | act (19.37, 1.0E-4) | extracellular vesicle (0.04) | 2009 |

The largest cluster (#0) has 130 members and a silhouette value of 0.761. It is labeled as  *apoptotic bodies* by LLR, *extracellular vesicle* by LSI, and *fate (0.25)* by MI. The most relevant citer to the cluster is BELLONE, M (2000.0) [Apoptosis, cross-presentation, and the fate of the antigen specific immune response](http://dx.doi.org/10.1023/A:1009671105696). APOPTOSIS DOI 10.1023/A:1009671105696.

The second largest cluster (#1) has 99 members and a silhouette value of 0.729. It is labeled as ***liquid biopsy*** by both LLR and LSI, and as *fate (1.32)* by MI. The most relevant citer to the cluster is YANG, Y (2018.0) [An immuno-biochip selectively captures tumor-derived exosomes and detects exosomal rnas for cancer diagnosis](http://dx.doi.org/10.1021/acsami.8b13971). ACS APPLIED MATERIALS & INTERFACES, V10, P12 DOI 10.1021/acsami.8b13971.

The third largest cluster (#2) has 91 members and a silhouette value of 0.715. It is labeled as  *cell-derived extracellular vesicle* by LLR, *extracellular vesicle* by LSI, and *fate (3.09)* by MI. The most relevant citer to the cluster is HUANG, W (2022.0) [Extracellular vesicles as theranostic tools in kidney disease](http://dx.doi.org/10.2215/CJN.16751221). CLINICAL JOURNAL OF THE AMERICAN SOCIETY OF NEPHROLOGY DOI 10.2215/CJN.16751221.

The 4th largest cluster (#3) has 78 members and a silhouette value of 0.716. It is labeled as  *ultracentrifugation supernatant* by LLR, *extracellular vesicle* by LSI, and *fate (2.16)* by MI. The most relevant citer to the cluster is SAENZ-PIPAON, G (2021.0) [Urinary extracellular vesicles for diabetic kidney disease diagnosis](http://dx.doi.org/10.3390/jcm10102046). JOURNAL OF CLINICAL MEDICINE, V10, P20 DOI 10.3390/jcm10102046.

The 5th largest cluster (#4) has 62 members and a silhouette value of 0.811. It is labeled as ***vascular calcification*** by both LLR and LSI, and as *fate (0.27)* by MI. The most relevant citer to the cluster is LONDON, G (2005.0) [Arteriosclerosis, vascular calcifications and cardiovascular disease in uremia](http://dx.doi.org/10.1097/01.mnh.0000168336.67499.c0). CURRENT OPINION IN NEPHROLOGY AND HYPERTENSION DOI 10.1097/01.mnh.0000168336.67499.c0.

The 6th largest cluster (#5) has 56 members and a silhouette value of 0.797. It is labeled as  *cell-derived extracellular vesicle* by LLR, *systemic lupus erythematosus* by LSI, and *fate (1.52)* by MI. The most relevant citer to the cluster is IKEYAMA, S (2000.0) [Apoptosis of proliferative cortical tubular epithelia in chronic progressive nephrosis of rats](http://dx.doi.org/10.1292/jvms.62.367). JOURNAL OF VETERINARY MEDICAL SCIENCE DOI 10.1292/jvms.62.367.

The 7th largest cluster (#6) has 42 members and a silhouette value of 0.779. It is labeled as ***urinary extracellular vesicle*** by both LLR and LSI, and as *mir-451 loaded exosome (0.22)* by MI. The most relevant citer to the cluster is ALASMARI, W (2022.0) [Exosomes derived from bm-mscs mitigate the development of chronic kidney damage post-menopause via interfering with fibrosis and apoptosis](http://dx.doi.org/10.3390/biom12050663). BIOMOLECULES, V12, P16 DOI 10.3390/biom12050663.

The 8th largest cluster (#7) has 23 members and a silhouette value of 0.959. It is labeled as ***crystal structure*** by both LLR and LSI, and as *extracellular vesicle (0.04)* by MI. The most relevant citer to the cluster is BOUMA, B (1999.0) [Adhesion mechanism of human beta(2)-glycoprotein i to phospholipids based on its crystal structure](http://dx.doi.org/10.1093/emboj/18.19.5166). EMBO JOURNAL DOI 10.1093/emboj/18.19.5166.

The 9th largest cluster (#8) has 18 members and a silhouette value of 0.95. It is labeled as  *cd95 activation* by LLR, *ion fluxe* by LSI, and *extracellular vesicle (0.04)* by MI. The most relevant citer to the cluster is REINEHR, R (2007.0) [Cd95 activation in the liver: ion fluxes and oxidative signaling](http://dx.doi.org/10.1016/j.abb.2006.12.019). ARCHIVES OF BIOCHEMISTRY AND BIOPHYSICS DOI 10.1016/j.abb.2006.12.019.

The 10th largest cluster (#10) has 4 members and a silhouette value of 0.997. It is labeled as  *act* by LLR, *oligonucleotides suppress pkb/akt and act as superinductors of apoptosis in human keratinocytes* by LSI, and *extracellular vesicle (0.04)* by MI. The most relevant citer to the cluster is KIPPENBERGER, S (2009.0) [Oligonucleotides suppress pkb/akt and act as superinductors of apoptosis in human keratinocytes](http://dx.doi.org/10.1093/nar/gkp252). NUCLEIC ACIDS RESEARCH, V37, P15 DOI 10.1093/nar/gkp252.
